# 3D Insights into the Effects of Captivity on Wolf Mastication and Their Tooth Marks; Implications in Ecological Studies of Both the Past and Present

**DOI:** 10.3390/ani11082323

**Published:** 2021-08-06

**Authors:** Lloyd A. Courtenay, Darío Herranz-Rodrigo, José Yravedra, José Mª Vázquez-Rodríguez, Rosa Huguet, Isabel Barja, Miguel Ángel Maté-González, Maximiliano Fernández Fernández, Ángel-Luis Muñoz-Nieto, Diego González-Aguilera

**Affiliations:** 1 Department of Cartographic and Terrain Engineering, Higher Polytechnic School of Ávila, University of Salamanca, Hornos Caleros 50, 05003 Ávila, Spain; mategonzalez@usal.es (M.Á.M.-G.); almuni@usal.es (Á.-L.M.-N.); daguilera@usal.es (D.G.-A.); 2 Department of Prehistory, Complutense University, Prof. Aranguren s/n, 28040 Madrid, Spain; dario.herranz.rodrigo@gmail.com (D.H.-R.); joyravedra@hotmail.com (J.Y.); 3 C. A. I. Archaeometry and Archaeological Analysis, Complutense University, Professor Aranguren 2/n, 28040 Madrid, Spain; 4 Department of Prehistory and Archaeology, Humanities Faculty, UNED University, C/Senda del Rey, 7, 28040 Madrid, Spain; jmvr.preh@gmail.com; 5 Institut Català de Paleoecologia Humana I Evolució Social (IPHES), Zona Educacional 4, Campus Sescelades URV (Edifici W3), 43700 Tarragona, Spain; rhuguet@iphes.cat; 6 Department d’Historia i Historia de l’Art, Universitat Rovira i Virgili (URV), Avinguda de Catalunya 35, 43002 Tarragona, Spain; 7 Unit Associated to CSIC, Departamento de Paleobiologia, Museo de Ciencias Naturales, Calle José Gutiérrez Abascal, s/n, 28006 Madrid, Spain; 8 Zoology Unit, Department of Biology, Autónoma University of Madrid, C/Darwin 2, Campus Universitario de Cantoblanco, 28049 Madrid, Spain; isabel.barja@uam.es; 9 Center of Investigation in Biodiversity and Global Change (CIBC-UAM), Universidad Autónoma de Madrid, 28049 Madrid, Spain; 10 Department of Topographic and Cartography Engineering, Higher Technical School of Engineers in Topography, Geodesy and Cartography, Universidad Politécnica de Madrid, Mercator 2, 28031 Madrid, Spain; 11 Gran Duque de Alba Institution, Dibutación Provincial de Ávila, Paseo Dos de Mayo, 8, 05001 Ávila, Spain; maxifernandezav@hotmail.com; 12 Department of Sciences of Communication and Sociology, Faculty of Communication Sciences, University Rey Juan Carlos, Camino del Molino, s/n, 28943 Madrid, Spain

**Keywords:** wild wolves, captive wolves, tooth marks, geometric morphometrics, 3D modeling, advanced statistics, taphonomy

## Abstract

**Simple Summary:**

Recent years have seen major advances in the analysis of carnivore modifications to bone during feeding, based on the integration of 3D modeling and data science techniques, and with special attention being paid to tooth marks. From this perspective, carnivore tooth scores and pits have slowly converted into a protagonist in the identification of the carnivores producing them. The present study confronts the intra-species variability of tooth mark morphologies produced by Iberian wolves, taking into account not only different populations but also whether wild and captive wolves produce different shaped tooth marks. Here we show how, in the case of tooth scores, differences are notable and should thus be treated with caution. Further conclusions reveal that carnivore tooth pits are currently the most diagnostic elements for the study of carnivore feeding traces on bone, pending future studies that compare closely related taxa with sufficient intraspecific variability. In light of this, further investigation into the possible stress captivity may cause on these animals could be of great importance for both the study of past and present. If differences were to exist, these results could implicate a larger margin of error than previously perceived for some experimental samples, affecting both prehistoric and modern-day ecological studies.

**Abstract:**

Human populations have been known to develop complex relationships with large carnivore species throughout time, with evidence of both competition and collaboration to obtain resources throughout the Pleistocene. From this perspective, many archaeological and palaeontological sites present evidence of carnivore modifications to bone. In response to this, specialists in the study of microscopic bone surface modifications have resorted to the use of 3D modeling and data science techniques for the inspection of these elements, reaching novel limits for the discerning of carnivore agencies. The present research analyzes the tooth mark variability produced by multiple Iberian wolf individuals, with the aim of studying how captivity may affect the nature of tooth marks left on bone. In addition to this, four different populations of both wild and captive Iberian wolves are also compared for a more in-depth comparison of intra-species variability. This research statistically shows that large canid tooth pits are the least affected by captivity, while tooth scores appear more superficial when produced by captive wolves. The superficial nature of captive wolf tooth scores is additionally seen to correlate with other metric features, thus influencing overall mark morphologies. In light of this, the present study opens a new dialogue on the reasons behind this, advising caution when using tooth scores for carnivore identification and contemplating how elements such as stress may be affecting the wolves under study.

## 1. Introduction

### 1.1. The Impact of Wolves Both Past and Present

Wolves are one of the most prominent carnivore taxa of the Northern Hemisphere, appearing in a number of ancient sites as a competitor with humans [1,2]. The genus *Canis* [3] has been most recently estimated to have emerged towards the Messinian stage of the Miocene, dated at approximately 5.7 Ma, with Bayesian inferred Highest Posterior Density (HPD) intervals between 8.5 and 4.0 Ma [4]. The evolution of *Canis* species variants has since had a long history, with the emergence of a wide array of different sized carnivores, from the smaller jackals (*Canis aureus*) to the Dire wolf (*Aenocyon dirus*) of North America. The species *Canis lupus* is considered to have emerged during the Middle Pleistocene, strongly linked with the evolution of species such as *C. mosbachensis, C. etruscus* [5,6], and the recently discovered *Canis borjgali* [7]. Thereafter, the *Canis* genus has had a notable presence in numerous sites throughout the Pleistocene epoch, closely coinciding with the movement of hominin populations across the globe, and in many cases coexisting with other competitors of the Canidae family, such as *Lycaon lycaonoides, Cuon alpinus* and *Canis orcensis* [5,8,9,10,11].

Canids have been an important protagonist within the European carnivore guild. To this extent, some authors hypothesize the trophic pressure and competition for resources among these species [10,12,13,14,15,16,17,18,19]. From a similar perspective, being a close competitor with large felids at the time, other studies hypothesize these canids to have had a dynamic role in complex food chains throughout the Pleistocene [1,2]. As of the Upper Palaeolithic, interactions between canid and hominin species begin to change, with possible evidence of domestication and collaboration as early as >30 Kya [20,21,22,23,24]. These interactions become more complex as the increase in human populations induces significant pressure on carnivores. From this perspective, animals such as wolves are frequently targeted by farmers and landowners as a means of protecting their livestock [25,26,27,28,29,30,31]. While wolves are typically the first suspects as culprits for livestock predation, formal clues to differentiate between potential predators are lacking (e.g., foxes, dogs), especially in cases where flesh evidence is scarce.

A recent study by Yravedra and colleagues [32] proposes the use of diagnostic criteria typically used in archaeology and paleontology for the study of carnivore activity. From this perspective, these authors propose methods on bone as a means of studying the agents responsible for livestock predation. From this perspective, these authors attempt to unify ecological studies of both past and present with the use of high-resolution technologies for 3D modeling and advanced statistics, as well as artificially intelligent tools. Nevertheless, while this line of research has made important advances in the differentiation between different species, little is known on the intra-species variability that may be conditioning these results. Examples of this could include changes due to sexual dimorphism, age differences, natural inter-individual variability, genetic differences between populations, or sufficient sample sizes that can capture these differences.

In archaeology and paleontology, the discerning of precise carnivore agents is a highly informative piece of information, essentially providing a fundamental means of interpreting carnivore-hominin interactions as well as the general ecology of many sites. In modern-day ecology, the conservation of carnivores is under threat from human activity, with livestock predation being a particular source of tension. Research into the types of damage carnivores can produce on bones can thus be considered an important means of finding diagnostic criteria and can therefore answer a number of different ecological questions (e.g., paleoecological modeling, discerning agents involved in contemporary livestock predation). Nevertheless, prior to any widespread applications, experiments must be performed to ensure that our comparative samples are truly analogous.

In light of this, the present study attempts to analyze the variability of tooth marks in wolf populations, testing for the effect of cofounding variables that had not been considered prior to the present study.

### 1.2. State of the Art in Wolf Tooth Mark Analyses

**Figure 1:**
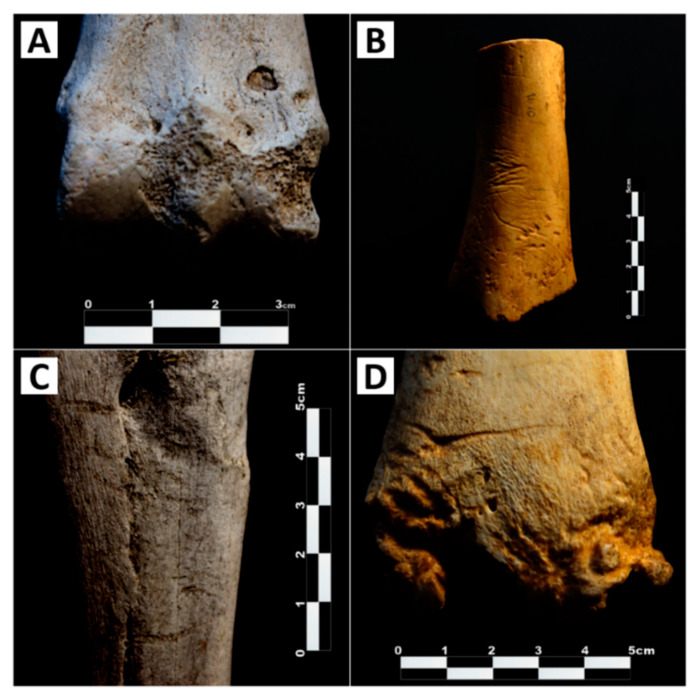
Photographic documentation of different types and the extent of bite damage. (**A**) Single isolated tooth pit observed on the distal metadiaphysis of a horse metatarsal from Villardeciervos. (**B**) Tooth-marked bone presenting multiple parallel scores across the diaphysis and pits towards the distal end of a horse humeral shaft from Cabárceno. (**C**) Multiple scores along the shaft of a horse radius-ulna from Villardeciervos. (**D**) Multiple scores and an occasional pit on the distal metadiaphysis of a horse tibia from Cabárceno.

**Figure 2:**
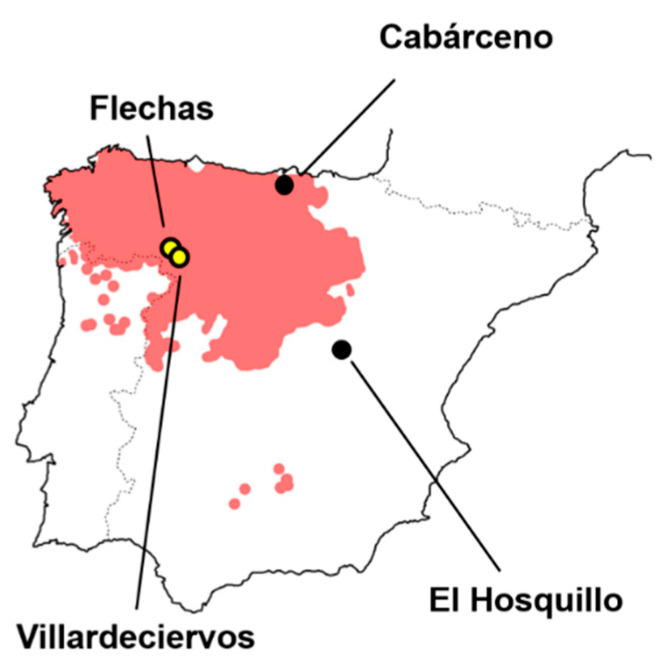
Graphic representation showing the distribution of wolves in the Iberian peninsula as well as the location of the wolf tooth mark sample used in the present study, including wild wolf packs (yellow dots) and captive wolves (black dots).

**Figure 3:**
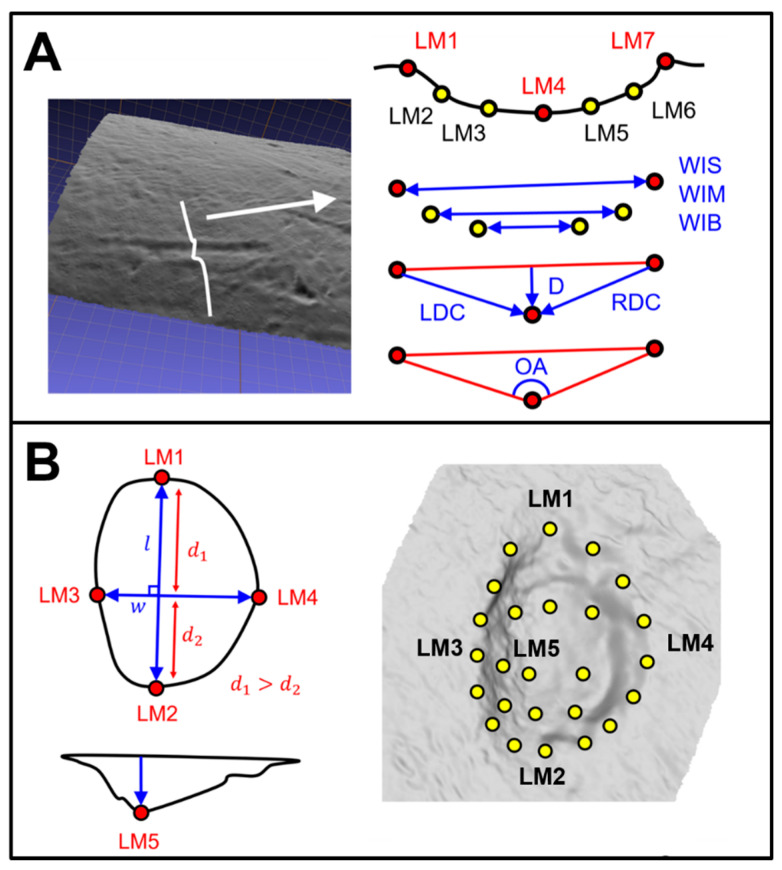
Visual description of landmark coordinate positions and the derived measurements. (**A**): 2D 7-Landmark model proposed by Yravedra et al. [40] with adaptations for the inclusion of equidistant computational landmarks by Courtenay et al. [53]. From the 7 landmarks, an additional 7 measurements can be derived, including the Width of Incision at Surface (WIS), Midway (WIM) and in proximity with the Base (WIB), alongside Depth (D), Left (LDC) and Right (RDC) Depth at Convergent and finally Opening Angle (OA). (**B**) 3D 30-Landmark model proposed by Courtenay et al. [46] with 5 fixed landmarks (Red) and a 5 × 5 computed landmark patch (yellow). The positioning of the fixed landmarks is dependent on the perpendicular axes that mark the maximum length (*l*) and width (*w*) of the pit, with Landmark 1 (LM1) being positioning the furthest away from *w* (distance 1, *d*_1_, must be greater than distance 2, *d*_2_).

Since the beginning of research into the faunal remains of archaeological sites, authors have noted the presence of large carnivore activity [33]. Among the different tracks and traces frequently left by carnivores, tooth marks found on bone have been considered of particular interest [34], providing an indirect trace of carnivore activity in sites where carnivore bones may not necessarily be present. The field of taphonomy is a particular protagonist in this type of research, focusing on the multiple agents and processes that may have modified bones since the life of the organism to the present date. At present, four main types of carnivore bite damage are typically considered [16,34,35,36]; (1) circular depressions or imprints of the tooth’s cusps on bone (tooth pits, Figure 1A); (2) elongated depressions with a rounded base produced by the dragging of teeth across the surface (tooth scores, Figure 1); (3) circular holes which are a product of the tooth penetrating the cortical walls (punctures)*;* and (4) the progressive deletion of large portions of bone by continuous chewing (furrowing).

Some of the first notable efforts in the study of tooth marks were performed by Selvaggio and Wilder [37], consisting of the metric analyses of the length and width of tooth scores and pits. While innovative, these methods present important limitations, discerning the most likely size of the chewing carnivore rather than inferring precise agents. More developed attempts were able to augment the size of available datasets, yet with similar limitations to their predecessors [38,39] (*inter alia*).

With the integration of advanced 3D modeling, novel efforts were able to improve this resolution, making closer approximations to the precise agent involved [40,41,42,43]. Despite the great improvement in statistical data processing techniques, these studies still presented margins of error that were hard to overcome. Likewise, while the inclusion of advanced data science techniques such as Computational Learning strategies was able to present a more efficient means of processing this data [44], these studies are still limited in terms of sample size and can only be considered the first step in a promising direction. From this point, more consequent efforts have been made to increase sample sizes with equally promising results [32,45] while also improving the means of extracting this information [46].

Other notable advances in literature have shown how volumetric and micro-topographical information derived from confocal microscopy can provide valuable information [47,48]. Likewise, deep learning-based computer vision techniques have also been proposed for answering a number of these questions [49,50].

While these studies are hopeful for the future of inter-species analyses, the intra-species perspective has been touched on less [51,52], especially from a morphological perspective [53]. In light of this, important questions must be raised prior to the large-scale application of these methods, such as the possible changes to tooth mark morphologies that may be a product of additional confounding variables. If these variables are found to be important conditioning factors, this may raise important doubts on the applicability of these techniques in real-world applications, on an inter-species as well as an intra-species level. This could essentially alter results in analyses such as the identification of carnivore activities in archaeological sites or the classification of animals responsible for livestock predation.

The present study considers these questions from the standpoint of modifications produced by multiple individuals of the same species; the Iberian wolf. Similarly, considering how obtaining bones modified by wild carnivores can be notably difficult, this study additionally tests to see whether using captive animals in parks is the best analogy for these types of modifications. The present hypotheses to be tested do not consider captivity to be a conditioning factor in tooth mark morphologies, while the difference between wolf populations should also be minimal. Under this premise, four large samples of bones modified by different populations of *Canis lupus signatus* individuals have been used, two originating from parks of varying sizes and the remainder of samples originating from wild wolf packs across the north-western Iberian peninsula.

## 2. Materials and Methods

### 2.1. Samples

For the purpose of understanding intra-species variability in carnivore populations, four samples of tooth-marked bones produced by *Canis lupus signatus* individuals were included in the present study (Figure 2).

*Canis lupus signatus* [54], commonly known as the Iberian wolf, is a subspecies of wolves populating the northwest of the Iberian peninsula. Wolves are social hunters with a diet predominantly consisting of large and medium-sized game. Wolves additionally present one of the highest potential bite forces for their size when compared with other large carnivores, with an estimated bite force of ≈1200 Newtons using the Carnassial teeth [55]. When chewing, most canids are known to show a preference for the use of teeth furthest back in the mouth, namely the posterior-most inferior molars and upper premolars/molars. A recent study has reported the larger cusp of these teeth to have a mean breadth of 13mm for female wolves and 14mm for male wolves in the case of lower molars, and 16mm for female wolves and 17mm for male wolves in the case of upper pre-molars [56].

Two of the present tooth mark samples originated from wild wolf packs residing in the province of Zamora in north-western Spain, sharing borders with Portugal and located north of the River Duero (Castilla y León, Figure 2). The first of these samples originated from the area of Flechas, while the second from Villardeciervos. Both samples were collected between the months of May and September of 2010, consisting of carcasses from a wide range of different animals, including equids (*Equus ferus*), red deer (*Cervus elaphus*), wild boars (*Sus scrofa*), and roe deer (*Capreolus capreolus*). Control of whether only wolves had intervened in samples was based on the current knowledge about the ecology of the area. In both samples, all anatomical elements were present (including the cranial, axial, and appendicular skeleton); however, only tooth marks originating from the diaphyses and meta diaphyses of appendicular long bones were considered. From Flechas, a total of 55 appendicular bone remains were recovered, from which a total of 63 tooth scores and 49 pits were used. From Villardeciervos, a total of 124 appendicular elements were recovered, from which a total of 56 scores and 79 pits were used. This results in a total sample size of 128 wild wolf tooth pits and 119 scores.

Tooth mark samples from captive wolves were obtained from two separate parks, including Cabárceno (Obregón, Cantabria, Figure 2) and Hosquillo (Cuenca, Castilla-La Mancha, Figure 2). Samples were collected during the winter months of 2010 and 2011. The first of these samples have been previously included in publications including [32,41,44,45,46,50,53,57], while the latter by [45,53,58]. Both samples were produced by groups of adult individuals, seven individuals in the case of Cabárceno and five in the case of Hosquillo. Animals were fed disarticulated limb elements with meat attached, while some occasional distal epiphyses of humeri and femora have been noted as still articulated with zygopodials. Bones were exposed to animals for varying periods of time, as established by the rules and conditions of each park, with Cabárceno wolves having access to the remains for a single week and Hosquillo individuals for three months.

The Hosquillo sample consists of mainly medium and small-sized animals, including mouflon (*Ovis musimon*), Iberian ibex (*Capra pyrenaica*), roe deer (*C. capreolus*), and wild boar (*S. scrofa*). The Cabárceno sample consists exclusively of large-sized animals; namely, equid (*E. ferus*) and some bovid (*Bos taurus*) remains. Once again, only tooth marks observed on diaphyses or meta diaphyses of appendicular long bone elements were considered. From Hosquillo, a total of 420 remains of highly fractured appendicular elements (femora, humeri, tibiae, and radii) were recovered, from which a total of 113 tooth scores and 113 pits were used. From Cabárceno, a total of 28 appendicular elements were recovered, from which a total of 56 scores and 42 pits were used. This resulted in a total of 169 scores and 155 pits from captive animals.

Both parks are dedicated to the conservation and investigation of different species, some of which are endangered, and keeping animals in open-air enclosures while also being open to the public for educational purposes. Nevertheless, in the interest of transparency it is important to point out that, while previous publications have referred to these samples as a product of semi-captive wolves, after careful evaluation and consideration of how wolves in the wild typically inhabit a large and variable territory with high mobility (≈10 km per day, with great variability among some reports [59,60,61,62,63,64,65,66], *inter alia*), we considered that the enclosures presented in each of these parks were too small to consider these animals as anything other than captive. Personal communications by ground keepers have reported the wolf enclosure in Cabárceno to have an extension of 2700m^2^, approximately 0.04% of the total area (740 ha) of the Cabárceno natural park, while the Hosquillo individuals have a slightly larger enclosure measuring 10,000 m^2^, approximately 0.1% of the 910 ha of the Hosquillo natural park. The Cabárceno natural park is a popular year-round destination housing a multitude of species, with approximately 600,000 visitors per year. The Hosquillo natural park, on the other hand, is considerably smaller, with a reported 14,426 visitors during the year 2015 and 20,000 in the year 2018, making it 97 to 98% smaller than that of Cabárceno. In the interest of understanding the behavior of these animals, we also consider it important to note that the Cabárceno natural park is accessible almost exclusively by motor vehicles, implicating that animals are surrounded by additional noise produced by cars on a daily basis. Hosquillo, on the other hand, while also being accessible by motor vehicles, provides a larger distance between the asphalt tracks and each enclosure in some areas while only being open to the public on weekends or festive days, with week days generally being reserved for scholarly activities, thus ensuring less exposure of the wolves to the public.

Finally, with regards to the Hosquillo and Cabárceno samples, wolves from both parks have been estimated to be five years old, with little difference in age between wolves from these parks.

While many more tooth marks of varying types were observed on these samples, the selected tooth marks were chosen on the basis of being found on diaphyses; elements more likely to survive extensive carnivore damage, as well as any other taphonomic processes frequently encountered in the fossil register. Marks were chosen based on the clarity of their micro-topography, while only isolated marks with no overlapping of traces were selected. Inconspicuous marks that would be difficult to model via remote-sensing techniques were also excluded.

All experiments involving carnivores were performed in accordance with the relevant guidelines as set forth by park keepers and general park regulations. No animals were sacrificed specifically for the purpose of these experiments. Likewise, carnivores were not manipulated or handled at any point throughout the collection of samples. In each of the parks, collection of chewed bones was performed directly by park staff, assisted by one of the authors (J.Y.). No licenses or permits were required to perform these experiments. In the case of Cabárceno and Hosquillo, bone samples were provided directly by the park in accordance with their standardized feeding protocols. For wild wolves, carcasses were collected by one of the authors (J.Y.), additionally aided by forest rangers where necessary. Once collected, all bone samples were cleaned in boiling water without the use of additional chemical agents.

Photographic examples of tooth pits and tooth scores from different samples have been included in Figure 1.

### 2.2. Data Collection

Tooth marks were digitized using the DAVID SLS-1 Structured-Light Surface Scanner located at the TIDOP Research Group of the Polytechnic School of Ávila (University of Salamanca, Spain), identical to the methods described in [46,53,67]. Once 3D models had been obtained for each of the tooth marks, marks were treated differently according to their type (Figure 3), following the methodological approaches by Yravedra et al. [40] with slight adaptations by Courtenay et al. [53] in the case of tooth scores, and Courtenay et al. [46] in the case of tooth pits.

Tooth scores were analyzed according to the morphology of their cross-sections (Figure 3), extracted at the mid-point of each score. For this, 2D data were derived from 3D models via the calculation of digital elevation models for micro-topographies. Cross-sections were thus extracted using the Global Mapper v.18 Geographic Information System (GIS) software, obtained at mid-length of each tooth score between ≈30% and ≈70% of the mark’s total length. Cross-section profiles were then exported as images for further processing in the tpsDig2 (v.2.1.7) software [68].

For each cross-section, 7-landmark 2D coordinates (x, y) are extracted, following the model proposed by Yravedra et al. [40]. Landmarks 1 and 7 (LM1 & LM7) mark the left and right shoulder of the scores’ cross-section, respectively, while LM4 marks the deepest-most point. LM2, LM3, LM5, and LM6 traditionally mark varying points along the marks’ walls. Nevertheless, these have recently been replaced by computational landmarks so as to avoid analyst subjectivity [53]. These computational points are therefore calculated at equidistant intervals by the tpsDig2 software: between LM1/LM4 for the left wall and LM4/LM7 for the right wall. These landmark coordinates can then be used to calculate the metric dimensions of each profile, inspired by the methods of Bello and Soligo [69], later adapted by Maté-González et al. [70] and Yravedra et al. [40]. From this perspective, the distance between LM1 and LM7 is used to calculate the Width of the Incision at the Surface (WIS) of the bone, while distances between LM2/LM6 and LM3/LM5 can be used to define the Width of the Incision Midway (WIM) and the Width of the Incision in proximity with the Base (WIB), respectively. The Depth of the mark (D) can then be defined taking the perpendicular distance between LM4 and the plane between LM1/LM7, while the distance between LM1/LM4 and LM7/LM4 is used to calculate the Left and Right Depth of the incision at Convergent (LDC and RDC). All the aforementioned measurements were recorded in millimeters. Finally, the Opening Angle (OA) of the mark is calculated in degrees (°) by triangulating LM1, LM4, and LM7, followed by calculating the interior angle of the LM4 corner.

For further analysis, each of the 2D landmark coordinates was also exported and formatted into morphologika files for the purpose of Geometric Morphometric statistical studies.

For tooth pits, the entire 3D morphology of marks was analyzed (Figure 3), using the 3D 30-Landmark model proposed by Courtenay et al. [46]. This landmark configuration consists of five fixed Type II landmarks and a 5 × 5 patch of computational landmarks. Of the five fixed landmarks, LM1 and LM2 mark the maximal length of each pit, while LM3 and LM4 are used to define the pit’s width. For correct orientation of the pit, LM1 can be considered to be the point along the maximum length furthest away from the perpendicular axis marking the maximum width. LM2 is thus the point marking the other extremity of the pit’s length. LM3 and LM4 then mark the extremities of the perpendicular axis, with LM3 marking the left-most point of the maximum width and LM4 marking the right-most point. LM5 is finally defined as the deepest point of the pit. The 5 × 5 computational landmark patch is then positioned over the entirety of the pit so as to capture the internal morphology of the mark. For tooth pits, landmark data collection was performed using the free IDAV Landmark Editor software (v.3.0.0.6), carried out by a single experienced analyst in accordance with the instructional video and Supplementary Appendix provided by the original publication of the landmark model [46].

Finally, attempts to adapt and extrapolate the 3D-based methods to the analysis of tooth score morphologies were tried and tested to see whether the resolution could be improved. Nevertheless, these experiments yielded limited results (Supplementary File S2; Table S2.1) due to the wide variability of tooth score micro-topographies. Under this premise, only the 2D-based method was used for tooth score analysis (See Supplementary File S2).

### 2.3. Statistical Analyses

#### 2.3.1. Metric Analyses

Metric data were subject to both univariate and multivariate analyses. Tests additionally were performed, separating the variable OA from other linear metrics (WIS, WIM, WIB, LDC, RDC & D).

Prior to any comparative hypotheses tests, homogeneity of data distributions were checked via multiple Shapiro–Wilks tests [71]. The hypotheses tests employed were then dependent on Shapiro–Wilk results, employing parametric tests when Gaussian distributions were detected and non-parametric tests upon rejecting this assumption. In each of the univariate cases, robust and traditional descriptive statistics were then employed [46,72,73,74], using the mean and median to report Gaussian and non-Gaussian central tendencies, followed by the first standard deviation or the square root of the Biweight Midvariance (BWMV; Supplementary File S3, Equations (S3.1)–(S3.3)). Two-One Sided equivalency Tests (TOST) were then employed to test for the magnitude of equivalency between samples according to Cohen’s *d* [75]. For parametric versions of TOST, Welch’s *t*-statistic was used [76]. In cases where non-parametric approaches were required, Yuen’s trimmed robust *t*-statistic was used [77,78]. For ease of differentiating between the two, from this point onward, non-parametric robust TOST is referred to as rTOST. It is important to note that, contrary to many other analyses of variance, both variants of TOST consider the Null Hypothesis (*H_0_*) to indicate differences between samples [79]. Equivalency testing was performed using the equivalence (v.0.7.2) R library.

In the case of circular metrics, a different approach was used, taking into consideration the trigonometric properties of angular data. Firstly, it is important to point out that on all accounts, the variable OA fits a *Von Mises* Distribution [80,81,82], thus conditioning the selection of descriptive and hypothesis tests employed (see Supplementary File S1). Under this premise, descriptive statistics included standardized kurtosis and skewness values [83], alongside sample circular variance as an evaluation of the symmetry of circular distributions. For the assessment of circular “normality”, two additional tests were considered. These included analyses of uniformity, using the Rayleigh test [84], and symmetry, using a robust reflective symmetry test [85]. Uniformity and symmetry consider the distribution around the entire circle; it is not the same to have a slight amount of skew as having an overall symmetrical distribution across the entire circle. From this perspective, the combination of these tests checks to see whether the distortion for each distribution is of importance.

Depending on the relative symmetry of the circular distribution, the central tendency *H_0_* was robustly calculated via either the mean (*θ-bar*) or median (*θ-tilde*) angle. Prior to any of these calculations, angles were converted into radians, and converted back to degrees only for the purpose of reporting the final results.

For two-sample hypothesis testing, three different approaches were used to assess differences and similarities between samples. These tests considered; (1) differences in mean, using a bootstrapped alternative to the Watson test [86]; (2) differences in median, using the randomized variant of Fisher’s non-parametric test [87]; and (3) differences in distributions, using the randomized variant of the Mardia–Whatson–Wheeler test [83,88]. In-depth descriptions of these analyses and the reasons behind their adoption can be consulted in the Supplementary File S1.

For multivariate analyses of these metric variables, dimensionality reduction via Principal Components Analyses (PCA) was performed. For PCA, the variable OA was combined with linear variables WIS, WIM, WIB, LDC, RDC, and D by prior transformations of OA into a new *xy* variable. This was carried out by first converting OA into radians, followed by the sum of linear transformations for each of these radian angles using cosine and sine calculations (*cosθ + sinθ*, see Supplementary File S1). This approach was chosen in light of the considerable decrease in dimensionality reduction error produced when including this transformed version of OA in PCA (Supplementary File S1, Figure S1.9). TOST and rTOST tests were then performed across the PC Scores to test for multivariate equivalence, using the top ranking PC scores explaining up to 95% of total sample variance.

Considering the frequent non-linear relationships between variables, PCA was complemented by non-linear dimensionality reduction technique to observe for possible differences in conclusions. For this purpose, the t-Distributed Stochastic Neighbor Embedding (t-SNE) algorithm was chosen, a popular technique used in Machine Learning [89]. t-SNE was performed using random initialization directly on each of the extracted measurements. This algorithm was trained for 500 iterations, using a perplexity parameter calculated as the ceiling of the square root of the sample size (n, i.e., ⌈√n⌉). t-SNE transformations projected data into a new ℝ^3^ feature space. t-SNE was performed using the Rtsne (v.0.15) R library.

Where considered complementary, two additional tests were performed. The first of these considered correlations between variables to better understand relationships metrics. For homogenous data, the parametric Pearson test was used [90], whereas inhomogeneous data were tested using the non-parametric Kendall τ rank-based test [91]. Finally, Bayesian Inferred effect sizes were also calculated according to Cohen’s δ [75], with the use of 95% High Posterior Density calculations on the posterior distribution. These were additionally accompanied by Probability of Superiority (PS) metrics. For Bayesian calculations, a Student’s t-distribution was used to infer the data’s distribution [92] robustly. For this analysis, the No-U-Turn (NUTS) extension of the Hamiltonian Markov Chain Monte Carlo (MCMC) algorithm was employed for sampling [93], as implemented in the PyMC3 (v.3.11) library of the Python (v.3.7.4) programming language. NUTS was fit using 4 MCMC chains, sampling using 1000 tuning steps and 5000 draw iterations. To avoid severe divergences of the NUTS sampling algorithm, a sampling step size of 0.9 was employed. This parameterization resulted in only three divergences throughout all samples (observed in the case of the variable D for wild wolves), an Effective Sample Size of >4000, good mixing across all trace plots, and an R-hat statistic of 1.0 for all parameters. Further details on the Bayes models are included in Supplementary File S4, including equations (Equations (S4.1)–(S4.4)) and a visual representation of the models in the form of a Kruschke diagram (Figure S4.1) [94].

Unless stated otherwise, all statistical applications were performed using the R programming language (v.3.5.3) and multiple packages.

#### 2.3.2. Geometric Morphometric Analyses

Geometric Morphometric analyses were performed first, including an orthogonal tangent projection and full Procrustes fit of landmark data [95]. This is a common technique in morphological analyses for data preparation and standardization. This process, frequently referred to as Generalized Procrustes Analysis (GPA), consists of multiple superimposition procedures (translation, rotation, and scaling) that aid in the quantification and visualization of minute displacements of individual landmarks in space. Nevertheless, considering observations made by Courtenay et al. [44,45,46,53], which reveal tooth mark size to be an important conditioning factor in morphological variance, GPA was then subjected to multiple allometric analyses to test for shape-size relationships [96]. For allometric analyses, multiple regressions were performed using the logarithm of centroid size to estimate shape, testing for the goodness-of-fit to conclude whether (1) shape is notably affected by size but also (2) to see if differences in shape-size relationships for different samples are present.

In cases where allometric relationships were concluded to be important, further geometric morphometric analyses were performed, excluding the scaling process of GPA. When allometry proved to be unimportant, a full GPA, including the scaling process, was used. This exclusion of the scaling process is often referred to as the analysis of *form*, while the inclusion of scaling during GPA is referred to as analysis of *shape* [97,98,99].

Following GPA, dimensionality reduction in the form of PCA was performed to convert landmark coordinates into a more manageable format. The most important Principal Component Scores (PC Scores) were then extracted for multivariate testing. Across these PC Scores, TOST and rTOST tests were carried out, alongside the calculation of transformation grids and Thin-Plate Splines (TPS) for the visualization of morphological changes [100].

Finally, considering the frequently non-linear relationships that have frequently been observed to emerge among taphonomic geometric morphometric data [46], PCA was also complemented by the use of t-SNE. In this case, t-SNE was trained directly on the superimposed landmark coordinates.

For all Geometric Morphometric analyses, the R programming language (v.3.5.3) was used, primarily employing the geomorph (v.3.3.1) and shapes (v.1.2.4) R libraries.

#### 2.3.3. Hypothesis Testing

In accordance with the recommendations set forth by the editors and contributors of the *American Statistician*, *p*-values were not evaluated using *p* < 0.05 as a threshold for defining statistical significance [101,102]. Likewise, the term “significant” has been avoided throughout the present study. In its place, all hypotheses testing was performed using *p*-value to Bayes Factor Bound (BFB) calibrations in accordance with the recommendations of Benjamin and Berger [103], as well as the calculation of False Positive Risk (FPR) values as suggested by Colquhoun [104]. BFB values (Supplementary File S5; Equations (S5.1) and (S5.2)) represent “the strongest case for the alternative hypothesis [*H_a_*] relative to the null hypothesis [*H_0_*]” [103], in other words, the odds at most of *H_a_* being true. These values can then be used to derive the final posterior odds by multiplying BFB with the prior odds. FPR (Equation (S5.3)), on the other hand, is the probability that an observed *p*-value is a false positive, otherwise known as a Type I error.

To avoid the inclusion of large strings of numbers in the main text reported values have been mostly limited to the inclusion of a single probability metric in support of the Null Hypothesis (*H_0_*), referred to here as the Probability of *H_0_*, or *p(H_0_)*, of the probability of error if *H_a_* were to be true (Equation (S5.5)). This can be calculated using a combination of FPR and an inverse function (Equation (S5.4)). Considering how Courtenay et al. [45] found the point of maximum curvature of calibration curves to be at *p* = 0.3681, *p(H_0_)* was defined here using this value as the optimal limit in Equation (S5.4).

All formulae used for these calculations have been reported in Supplementary File S5 (Equations (S5.1)–(S5.3)), alongside additional calibration tables (Tables S5.1–S5.4) and curves (Figure S5.1). Precise BFB and FPR values for a selection of *p*-values can be consulted in Tables S5.1–S5.4. Noting the poor quality of *p* < 0.05 as a boundary for strong evidence of *H_a_*, the present study chose to adapt Fisher’s [105] definition of the second standard deviation from the mean (i.e., 0.05), extending this to the third standard deviation from the mean (100% − 99.7% = 0.3% or 0.003). Finally, unless specified otherwise, all frequentist to Bayesian calibrations have been performed using a prior probability indicative of complete randomness (0.5), as suggested by Colquhoun [104].

For more details on the present use of BFB and FPR values, consult [103], ref [104], or the summary reported in Supplementary Appendix 3 of Courtenay et al. [45]. For more details on *p(H_0_)* consult Courtenay et al. [106]. The code used to perform these calculations can be obtained from https://github.com/LACourtenay/HyperSkinCare_Statistics (accessed on 05/08/2021) [106].

## 3. Results

### 3.1. Morphometric Analyses

#### 3.1.1. Analyses of Opening Angles (OA)

Opening Angles presented a mixture of “normally” and “abnormally” angular distributions (Table 1); furthermore, wide variability was observed, with captive wolves presenting more obtuse (*θ-tilde* = 151°) and variable (*v* = 0.03) tooth scores than wild wolves.

General analyses of differences and similarities among OA results show relatively clear differences between most samples (Table 2), with the only exception being comparisons between both wild wolf samples. The greatest magnitude of differences, however, when considering both calculations for mean, median and overall distribution, are found between wild and captive wolves (test-statistics > 69.7, *p* < 0.0001, *p(H_0_)* < 0.25%), especially in the case of the Cabárceno captive and Villardeciervos wild wolf samples (test-statistics > 55.3, *p* < 0.0001, *p(H_0_)* < 0.25%).

#### 3.1.2. Analyses of Measurements

Linear measurement values show highly inhomogeneous distributions on all accounts (W > 0.87, *p* < 3.7 × 10^−06^, *p(H_0_)* < 0.01%). Univariate tests reveal important similarities for all metric variables obtained from different wolf populations (rTOST |*d*| < 0.136, *p* < 0.003, *p(H_0_)* < 4.5%), with the exception of tooth score depth (|*d*| ≈ 0.042, *p* ≈ 0.658, *p(H_0_)* ≈ 57.2%). The magnitude of these differences in D increases when considering samples according to captivity (|*d*| = 0.028, *p* = 0.856, *p(H_0_)* = 73.4%), indicating overall differences in tooth pits to be more likely conditioned by captivity than differences in wolf populations. Moreover, when considering tooth score widths (WIS), similarities are minimal (|*d*| = 0.127, *p* = 0.145, *p(H_0_)* = 43.2%).

In light of these observations, and considering each of the descriptive statistics for the samples in Table 3, it can be seen that the largest differences between samples are found in the depth of tooth scores, with captive wolves producing marks approximately 0.4 +/− 0.2 mm shallower than wolves found in the wild. Furthermore, clear relationships can be established between depth and width (Kendall’s τ = 0.59, *p* < 2.2 × 10^−16^, *p(H_0_)* < 2.2 × 10^−12^%), as well as OA (Kendall’s τ = −0.5, *p* < 2.2 × 10^−16^, *p(H_0_)* < 2.2 × 10^−12^%). From this perspective, the marked increase in wild wolf tooth score D values is likely to condition the increased variability and change in tooth score widths (Table 3; Captive √BWMV = 0.33, Wild √BWMV = 0.27). Nevertheless, the magnitude of these differences according to Cohen’s δ are relatively small for WIS (δ = 0.26, PS = 0.57), with Bayesian inferred differences of 0.08mm in central tendencies (95% HDI = [0.015, 0.017]), while differences are much larger for D (δ = 0.77, PS = 0.71), with differences of 0.048mm in central tendencies (95% HDI = [0.019, 0.020]).

Coupled with previous observations regarding the notably larger opening angles of these scores (Table 1), it can be predicted prior to any multivariate or geometric morphometric tests that the overall morphology of tooth scores will be different between wild and captive wolves.

**Figure 4:**
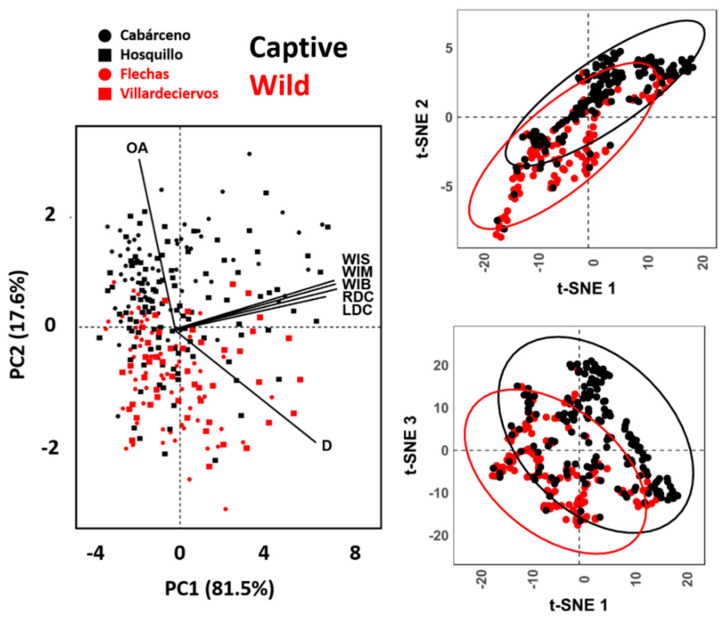
PCA scatter-biplots and t-SNE scatterplots performing dimensionality reduction on metric variables obtained from tooth scores.

When combining all 7 variables multivariately, both PCA and t-SNE (Figure 4) show clear separations between wild and captive wolves, while populations intermingle with no clear patterns. t-SNE results show high clustering of groups, with occasional overlap across all three dimensions. When considering PCA, the first PC Score (PC1) representing 81.5% of sample variance and is represented primarily by variables WIS, WIM, WIB, LDC y RDC, while the second component is strongly conditioned by the prior-mentioned patterns between D and OA, representing 17.6% of the variance. PCs 3 through to 7, however, are mostly residual (Cumulative variance = 0.9%). rTOST results strongly confirm previous observations, highlighting the greatest magnitude of differences to occur across PC2 (D & OA; |*d*| = 0.85, *p* = 0.99, *p(H_0_)* = 97.4%), while differences in tooth score width measurements across PC1 are much smaller, yet still fairly conclusive (|*d*| = 0.15, *p* = 0.146, *p(H_0_)* = 43.3%).

### 3.2. Geometric Morphometrics

#### 3.2.1. Allometric Analyses

**Figure 5:**
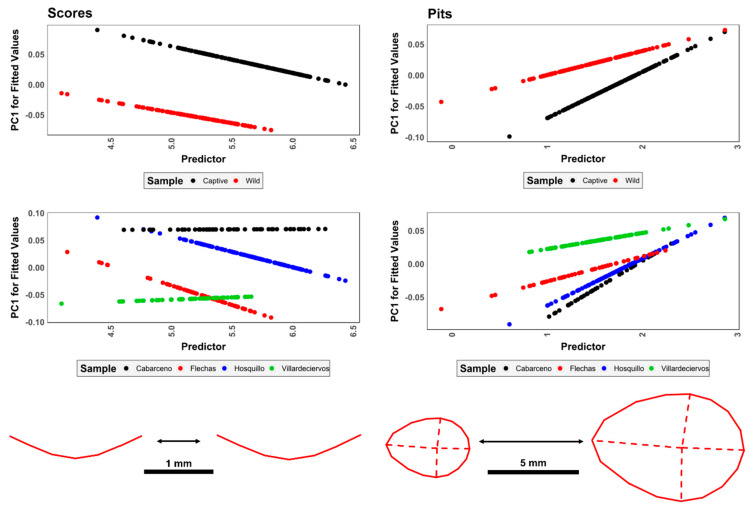
Visualization of shape allometry, mapping out tooth mark morphology as a function of size. Predicted values (ŷ) are sample-specific (Upper panels: captivity vs. wild; lower panels: population origin) in combination with logarithmic centroid size.

Shape-size relationships and simple allometric patterns reveal fairly strong tendencies for size to influence morphology in the case of pits (F = 2.8, *p* = 0.009, *p(H_0_)* = 10.3%), while scores tend towards the contrary (F = 2.627, *p* = 0.082, BFB = 1.79, *p(H_0_)* = 35.8%) (Figure 5). Nevertheless, variations in results begin to appear when calculating differences across samples according to groupings.

Scores present inconclusive shape-size relationships when considering variables such as captivity (F = 0.23, *p* = 0.8, *p(H_0_)* = 67.3%), however studies regarding populations argue otherwise (F = 3.15, *p* = 0.01, *p(H_0_)* = 11.1%). In-depth analysis of tooth-score residual data, however, presents strong deviations from normality (w > 0.87, *p* < 2.0 × 10^−12^, *p(H_0_)* < 1.5 × 10^−08^%), with a high level of standardized residual disbalance across both Euclidean distances and fitted values. This is especially relevant considering residuals when plotted against the first principal component (w = 0.88, *p* = 8.9 × 10^−15^, *p(H_0_)* = 7.8 × 10^−11^%, |skewness| = 1.4), and predicted values through regression models (w = 0.87, *p* = 5.8 × 10^−15^, *p(H_0_)* = 5.2 × 10^−11^%, |skewness| = 1.48). Additionally, considering the irregular residual spread with no concentration around the line of best fit (|kurtosis| = 2.5), regressions estimating linear relationships between logarithmic centroid size and morphological variance according to samples are unlikely to detect true relationships efficiently.

While the *p* = 0.01 for population differences in scores is indicative of allometry, this *p*-Value corresponds to an upper bound on the Bayes factor of 7.98, which combined with diffuse prior odds of 1:2, would imply posterior odds of 3.99 in favor of the alternative hypothesis and an 11.1% chance of being a false positive. Nevertheless, considering the important levels of residuals produced by these models, it can be assumed that some of these relationships are being exaggerated by the parametric nature of the regression model. In order to adjust for this bias, it could be argued that a more conservative prior probability of 3:10 be adopted. Under this premise, the false-positive risk increases to 22.6%, associated with posterior odds of 2.40 in favor of the alternative hypothesis, and thus presenting a corrected *p(H_a_)* value of 77.4% (1- p(H_0_)). From a critical perspective, it can thus be argued that shape-size relationships according to scores be inconclusive based on the present data, especially when withdrawing finite conclusions according to samples.

When considering pits (Figure 5), unimportant relationships are revealed for both captivity (F = 0.7, *p =* 0.62, *p(H_0_)* = 55.4%) and population (F = 1.3, *p* = 0.139, *p(H_0_)* = 42.7%), with highly notable deviations from normality when considering residual data as well (w > 0.83, *p* < 2.6 × 10^−09^, *p(H_0_)* < 1.4 × 10^−05^%). Nevertheless, residual data for pits present a much stronger concentration around the line of best fit (|kurtosis| = {4.55:4.59}), alongside a more even spread across fitted values.

When exploring other possible noise generated within the samples and revisiting the effects observed when studying prey size [53], the present study is able to consolidate the argument that animal size is not the conditioning factor for the morphological variation observed within these samples (Figure 5; F = 1.17, *p* = 0.25, *p(H_0_)* = 48.5%). Nevertheless, strong allometric correlations do begin to emerge when considering tooth scores from the Cabárceno (*p* < 2.2 × 10^−16^, *p(H_0_)* < 2.2 × 10^−12^%) and Hosquillo (*p* < 2.2 × 10^−16^, *p(H_0_)* < 2.2 × 10^−12^%) samples separately. Under this premise, additional tests were performed using metadata obtained from both parks to analyze the effects of captivity-related stress. For these analyses, stress was modeled considering the number of individuals kept in the space provided by each park as well as the number of individuals in said space. Important correlations were detected (*p* < 2.2 × 10^−16^, *p(H_0_)* < 2.2 × 10^−12^%), resulting in an equally strong effects on morphology (F = 3.1, *p* = 0.001, *p(H_0_)* = 1.8%). While residuals remain distinguishably high (w = 0.91, *p* = 7.8 × 10^−09^, *p(H_0_)* = 4.0 × 10^−05^%), adopting a rigorous prior probability of 3:10 still reveals only a 4.2% probability of these observations being a false positive, posterior odds of 15.98 in favor of the alternative hypothesis, upper bound Bayes Factor of 53:1 against *H_0_*_,_ and thus a corrected *p(H_a_)* of 95.8%.

From this perspective, data from the present study indicate that stress may be an important conditioning factor in tooth score morphology, an observation that warrants further in-depth investigation.

#### 3.2.2. Analysis of Variance

When analyzing tooth mark variability in pure morphological shape space, scores continue to appear to present notable differences between wild and captive wolves. This is once again confirmed by very large magnitudes of difference across the first 3 PC scores (96.0% variance, |*d*| = 0.15, *p* = 0.99˙, *p(H_0_)* = 99.99%). When considering these differences in accordance with wolf populations, all samples present morphological differences (Table 4).

Tooth pits, on the other hand, produce PCA graphs of a much different nature, with form space revealing unimportant differences between wild and captive wolves across the first 5 PC scores (86.77% of variance; |*d*| = 0.13, *p* = 9.0 × 10^−14^, *p(H_0_)* = 7.4 × 10^−10^%). Even if considering a strict prior probability of 3:10, the worst-case scenarios present a probability of 1.7 × 10^−11^% of Type I statistical errors. For populations (Table 5), the same can be said throughout comparisons.

**Figure 6:**
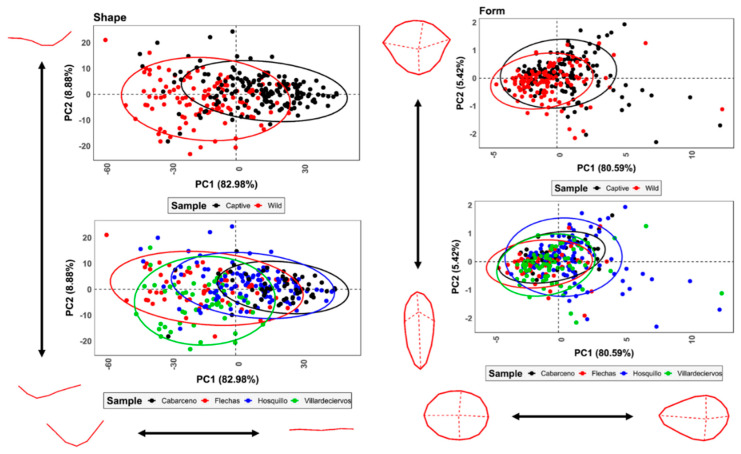
PCA scatter plots with 95% confidence intervals presenting variance in tooth score and tooth pit morphology, as represented in shape and form space, respectively. Morphological variance calculated through grid warpings is presented at the extremity of each PC score.

**Figure 7:**
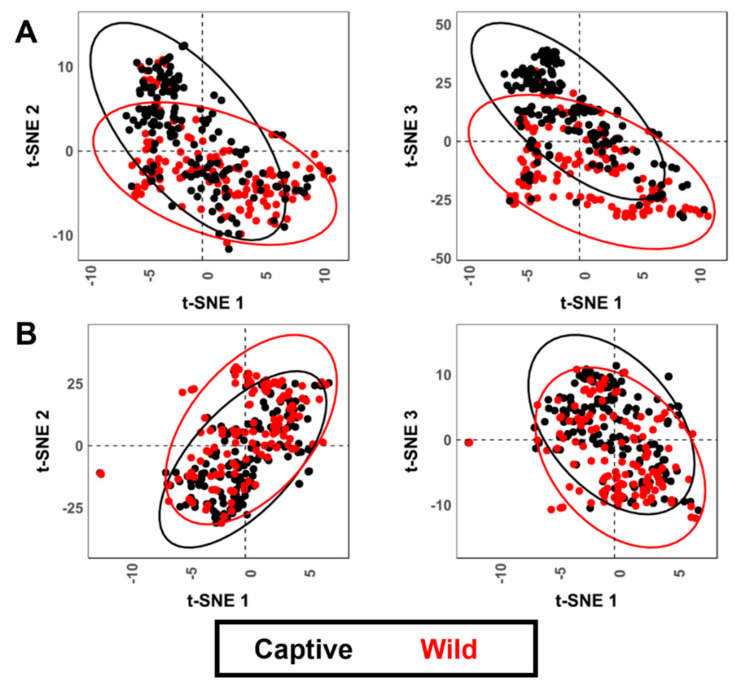
t-SNE scatter plots for both tooth (**A**) scores and (**B**) pits when performed analyzing shape and form variables, respectively.

Upon analyzing projections and morphological variations (Figure 6), Thin-Plate Splines (TPS) confirm that tooth scores are greatly represented by a shift from deep grooves in the case of wild wolves (83% of variance) while tooth pits appear much more complex. Tooth pits show high degrees of overlap throughout while revealing general tendencies for both wild and captive wolves to produce between circular and ovular pits on numerous occasions.

t-SNE results (Figure 7) for both cases similarly show very high clustering of tooth scores according to group labels, while tooth pits show no clear trends.

**Figure 8:**
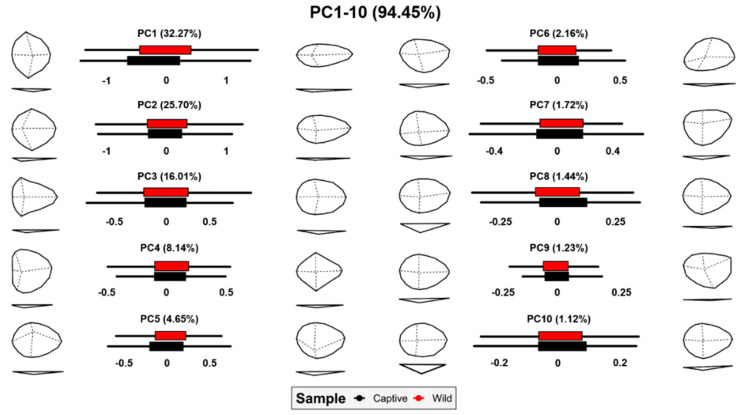
Box plots and morphological variance calculated through grid warpings for each of the dimensions in tooth pit PCA.

Finally, and in light of observations regarding wolf scores, a detailed analysis of morphological variations was performed across the first 10 PC scores (Figure 8). As can be seen, tooth pits present high morphological variability described by a number of features. Depth of tooth pits does not appear to be a conditioning factor until at least PC8, while only representing 1.44% of morphological variation. While it is true that wild wolves can be seen here to produce deeper pits than their captive relatives, geometric morphometric data highlight tooth pits to be better represented by a number of other morphological factors, as opposed to solely their depth.

## 4. Discussion

The conservation of any animal species requires an in-depth understanding of their behavior and ecology. The techniques, therefore, required to analyze animal activities are fundamental. Over the years, archaeologists and paleontologists have developed tools and techniques for the analysis of carnivore activities. While the objectives and research questions from these fields greatly differ from those of modern-day ecological studies, many parallels exist that could benefit from a more transdisciplinary approach to carnivore research.

The present study has shown through both metric and geometric morphometric approaches how wolf tooth scores show important differences when samples are obtained from captive animals. From this perspective, wolf tooth pits have been seen to be more diagnostic elements, less affected by inter-species variability, and still a valuable tool for intra-species analyses [45,46]. The discovery that wolf tooth marks are more superficial among captive wolves raises a number of questions, especially regarding their interpretation. Under this premise, the following sections attempt to describe and contemplate the possible conditioning factors that are behind these observed patterns in variance.

### 4.1. Interpretations behind Morphological Variability

#### 4.1.1. Physiological Stress of Captivity

Imposing captivity on wild animals has a number of functions. From one perspective, many institutions offer shelter, protection, and health care to species endangered by numerous external environmental factors (most of which are human-induced) and can also serve an educational purpose. In the latter case, this may be for the purpose of investigation while also serving the purpose of providing basic information to the general non-specialized public. Needless to say, captivity can have a major impact on an animal’s behavioral, social, or even physiological attributes. One of the key components involved in these processes is known as physiological stress [107].

Stressors may include but are not exclusive to the presence or absence of sensory stimuli (e.g., sound, smell, light, temperature), restrictions of movement (e.g., difficulties to retreat, forced proximity), abnormal social contexts (lack of possibilities for migration, forced husbandry), and the forced imposition of routine (feeding times, type of food, predictable presence or absence of stimuli). Nevertheless, despite the possible combinations of stressors, what is most likely to produce stress among captive animals is their inability to control them [107,108]. In the case of sensory stimuli, most animals, when exposed to uncomfortable situations, have the ability to flee, thus establishing control over the situation. While many zoos and parks housing captive animals pay particular attention to the design of enclosures, the impact of these factors is inevitable [109], especially over prolonged periods of time.

In response to these stressors, captive animals are well known to develop Abnormal Repetitive Behaviours (ARBs). While, in some cases, ARBs are interpreted as a coping mechanism, many specialists argue that this is not necessarily the case [110]. The severity and extent of these ARBs can be dependent on multiple factors; however, they are present in most, if not all, captive species. From one perspective, Mason [111] and Mason et al. [108] state that highly migratory animals are more likely to develop negative ARBs. From a similar perspective, these authors describe how a species’s plasticity and socio-cognitive attributes are likely to determine how well these animals cope under stress. Nevertheless, ARBs can not only be seen as an indicator of welfare quality and animal adaptability but, if too extreme, can also put into question the educational objective of these institutions, as these behaviors are not a true reflection of the wild animals’ original behavior.

A study by Wells [112] described how correlations exist between the aggressive actions of captive primates and the number of visitors to the zoo. While the social and cognitive attributes of primates may be a poor analogy to that of wolves, wild canids are still openly sociable animals with particular tendencies to interact with, or at least be acutely susceptible to, human-induced stimuli [113]. Likewise, studies carried out on smaller canids show an important change in fox behavior related to the higher peaks in visitors [114]. In each of these cases, the forced proximity with humans is likely to trigger different reactions among animals, which can be perceived as either a threat or a simple annoyance depending on the species [107].

Wild wolves typically modify the extension of their territories in order to avoid other wolf packs but are also frequently found to make particular efforts to avoid humans or human-made structures [66]. A study by Clubb and Mason [115], with the subtitle “Animals that roam over a large territory in the wild do not take kindly to being confined”, found correlations between natural territoriality and most ARBs in a number of different animals. From this perspective, and given the reduced amount of “territory size” provided by both the Cabárceno and Hosquillo centers, the size of enclosures and number of visitors are likely to have a joint impact on wolf behavior.

From another perspective, animals tend to eat when they need to, while the added removal of the thrill of the hunt is likely to agitate carnivores [116]. Likewise, the added security of being fed regularly is likely to affect their physiological stress levels.

Finally, chewing is known to calm domestic dogs when they get agitated [117]. Domestic dogs suffering from confinement distress have also been known to chew and destroy items, the latter more common when combined with noise aversion [118,119]. From this perspective, it is a well-known phenomenon that canine species are more prone to noise-related stress, with sounds such as gun-shots, fireworks, people yelling, and heavy traffic causing many dogs to react negatively. Destructive behavior, for example, is one of the most common reactions of dogs in these situations [118]. While many dog species are more accustomed to gun sounds, desensitized to loud noises, and are thus generally less prone to destructive behavior, this is a product of gradual introduction to these experiences, and in some cases, have become a genetic characteristic developed over time [120].

Closely related to some of these points, the lack of new stimuli in most captive environments is a major component in animal temperament. In more general terms, this can be summarized in the simple term “boredom”, and is especially relevant in animals of naturally high mobility confined in small spaces [115]. While canids are frequently known to chew on objects to relieve stress, this can sometimes be referred to also as a pass-time and is common, especially in bones found from wild wolf dens or domestic dog yards/kennels [16,35,121]. Analogies of ARBs in taphonomic analyses are commonly known, as in the case study presented by Gidna et al. [51], who found that the number of tooth marks left by captive lions (from Cabárceno, Spain) was almost twice the amount than those left by wild lions (Tarangire, Tanzania). A comparable study by the same authors showed leopards to produce even more extreme differences in the case of captive (Bahari Zoo, Dar es Salaam, Tanzania) and wild (Tarangire, Tanzania) leopards [52]. Likewise, studies by Saladié et al. [122] with captive bears originating from the Barcelona Zoo and Hosquillo Park present much higher tooth mark frequencies than data presented by Sala and Arsuaga [123] and Arilla et al. [124], with wild bears from Cantabria (Spain) and areas of the Spanish Pyrenees. While a full taphonomic analysis of the samples at hand is beyond the scope of the present study, similar observations have been made here (Supplementary Tables S6.1–S6.3).

In each of these examples, evidence points towards captivity being a stressful situation for animals; whether this physiological stress is enhanced by the presence of noise, crowds, lack of space, lack of external stimuli, or a combination of all of the above. In the case of Cabárceno and Hosquillo, both wolf groups are limited to a considerably smaller amount of space than they would experience in the wild. Moreover, this small space is shared between five to seven wolf individuals. Both samples also originate from parks frequently visited by large numbers of people, all of whom carry out their visit by motorized vehicles. Both of these factors are accompanied by the associated noise of both the crowds and the engines. Finally, alongside the established routines and lack of external stimuli, wolves are likely to present signs of boredom, which is commonly associated with excessive chewing.

While it could be hypothesized that the combination of these factors might cause wolves to bite harder, leaving deeper pits, if these continuous chewing behaviors persisted over long periods, this ARB might cause cusps to wear down through excessive use. This would explain the shallower marks. Nevertheless, further research would be required in order to prove this point.

#### 4.1.2. The Biomechanics of Mastication & Additional Reflections on Courtenay et al. “The Effects of Prey Size on Carnivore Tooth Mark Morphologies”

Multiple factors are involved behind the mechanics of mastication, many of which are highly complex, having evolved over millions of years. A previous study by the present authors performed analyses on how tooth mark morphology may be dependent on prey size [53]. Said study statistically concluded that evidence was “insignificant”, especially in the case of tooth pits. The discussion of the said study made reference to the influence of skull morphology, tooth morphology, and muscular functions, as well as attributes concerning lifestyle (see discussion of Courtenay et al., [53] and citations therein). While some of these conditioning factors are still to be experimented with, the present study provides a much larger sample size, providing a more empirical means of responding to some of these questions.

First, considering how the aforementioned behavioral attributes behind “playing” and “feeding” are likely to produce different biomechanical movements, it is likely that this is a notable change in the way force is exerted. From another perspective, comments by Toledo–González et al. [56] note that sexual dimorphism is an important component in wolf dental attributes. While controlling the intervention of different sexes in tooth marked samples is difficult, especially in the case of wild wolf samples, the lack of intra-group clusters and detectable patterns in each of the projected feature spaces (Figure 6) is likely to imply that this is not a significant conditioning factor in the case of tooth pits and scores.

In hindsight, and in light of the present study’s more rigorous use of *p*-values [103,104], it can be argued that in the case of tooth scores, the probability of these observations being a Type I statistical error falls to 30% for *Large* vs. *Small*-sized prey, 11% for *Large* vs. *Medium*-sized prey, and 8.7% for *Medium* vs. *Small*-sized prey. For tooth pits, Type I statistical error probabilities are between 25 and 4%, with the former calculation being relevant to tooth marks on large-sized animals. From this perspective, the difference between tooth scores on *Large* and *Small*-sized prey as originally reported [53] is likely to be an overestimate. Likewise, tooth pits show larger animals to be more problematic. When paired with a much larger sample here, comparing different populations, these patterns notably decrease. Once again, tooth marks in both wild wolf samples present a lack of separation between different sized animals, with an even smaller probability of being a False Positive for tooth pits (new *p* = 3.2 × 10^−^^18^, FPR = 3.5 × 10^−16^%). Likewise, when compared in a broader context across an additional 144 tooth marks, allometric relationships are revealed to be unimportant when considering prey size (Figure 5; F = 1.17, *p* = 0.25, *p(H_0_)* = 48.5%). From this perspective, the present study can thus consolidate previous hypotheses and conclude with more certainty that an animal’s prey size is not a powerful conditioning factor.

### 4.2. Implications for Carnivore Based Research

The present study has provided new insights into the effects captivity may have on typical carnivore modifications to bone, supporting and contributing to the discussions proposed by Gidna et al. [51] and Sala et al. [125]. Here we have found that tooth scores are notably different, while tooth pits are more dependent on other morphological features, leading captivity to be an unimportant conditioning variable. Nevertheless, to what extent are these observations likely to affect other research using carnivore tooth scores?

At present, multiple different techniques exist for the study of carnivore tooth marks, from more traditional metric variables based on simple measurements [37,38,39], to more complex means of extracting these measurements via 3D models and micro-topographies [40,41,46,47,48], as well as visual elements extracted from images [49,50]. The present study paid particular attention to how captivity affects variables such as WIS, which can be considered an analogy with any of the metric variables described by authors for the metric study of scores [37,38,39]. Likewise, the evident changes to score depth, and in turn, the correlated effect this has on WIS, WIM, and WIB variables, surely affects the volumetric properties of these marks [47,48]. From a multivariate approach and considering overall morphology, this has an evident impact on how tooth scores can be studied in geometric morphometric approaches as well [40]. While the present study has not directly considered qualitative components, the aforementioned effects of metric variability are also likely to condition the appearance of these traces in some way or another [49,50].

As noted by Clubb and Mason [115], the ability of a carnivore to adapt to captivity is greatly conditioned by their natural mobility and territoriality in the wild, as well as other factors, including behavioral and physiological attributes [107,108]. Wolves are well known for their high mobility [59,60,61,62,63,64,65,66], and therefore, are more likely to be susceptible to stressors. Nevertheless, the way a wolf deals with this stress may be different from that of other animals. Felids and ursids, for example, have been observed to develop ARBs in the form of extensive pacing [110]. Likewise, these animals are much less durophagous, therefore can be assumed to be less likely to modify bones to the same extent, while truly durophagous animals such as hyaenids have dentition specifically evolved for these types of repetitive forces [126]. This hypothesis, however, is yet to be supported by empirical evidence.

Mindful of all these elements, the present results advise caution when working with tooth scores, especially in the case of wolves. Until further experimentation has been carried out on felids, ursids, and hyaenids, as well as other canids, care must also be taken. Finally, it is important to encourage caution for taphonomists and analysts when publishing samples using the terms “semi-captive”, as this term can be considered misleading. The term “semi” should thus consider a number of different factors, including the general size, physiology, and natural behavior of the animal in the wild while factoring in the conditions under which the animals are enclosed.

## 5. Conclusions

The present study started with the hypothesis that, regardless of the population or state of captivity, tooth mark morphologies among wolf populations would not vary. While this hypothesis has been confirmed in the case of tooth pits, tooth scores have presented a notable deviation from our original theories.

The present study has shown a wide array of different perspectives on the effect captivity may have on the morphology of tooth marks. The importance of these observations has an impact on both past and present ecological studies. In conclusion, tooth scores have been shown to present notable variability and should thus be approached with caution when used as a diagnostic tool in tooth-marked assemblages. Nevertheless, pits are less dependent on these variables and are more likely to be a more reliable diagnostic tool for carnivore identification. Future investigation should take into account further interspecific analyses.

## Figures and Tables

**Table 1:** Descriptive data for tooth score opening angles. *k-hat* = standardized kurtosis, *s-hat* = standardized skew, *v* = Sample circular variance, t = test statistic, *p* = *p*-value. Bold typeface indicates samples where robust statistical measurements have been used. More details about reported values can be found in Supplementary Materials. *p(H_0_)* values have been excluded from the present table due to space restrictions. General calibrations for these values can thus be consulted in Supplementary File S5.

Sample	Min ^1^	*k-Hat*	*s-Hat*	*v*	Uniformity		Symmetry		Central ^1,2^	
					t	*p*	t	*p*		
Cabárceno	120.73	2.28	2.28	0.02	0.98	1.1 × 10^−23^	1.63	0.110	158.67	176.39
Hosquillo	112.00	0.25	1.06	0.02	0.98	1.2 × 10^−46^	1.77	0.079	147.09	175.12
Flechas ^3^	101.65	−1.31	−0.14	0.03	0.97	3.1 × 10^−26^	0.24	0.810	132.51	160.28
Villardeciervos ^3^	110.80	−1.49	0.41	0.01	0.99	6.3 × 10^−24^	0.75	0.447	132.19	149.96
Captive	112.00	0.27	1.32	0.03	0.97	3.2 × 10^−69^	2.60	0.007	151.40	176.39
Wild	101.65	−0.96	−0.03	0.02	0.98	2.0 × 10^−49^	0.06	0.953	132.36	160.28

^1^ Values reported in degrees (°). ^2^ Central tendencies are measured as a mean (θ-bar) or a median (θ-tilde) depending on whether non-robust or robust statistical measurements were used. ^3^ Wild animal samples.

**Table 2:** Statistical comparisons of Opening Angles between samples testing for common mean, median, and distribution. *Y*_g_, *P*_g_, and *W*_g_ are the test statistics for the corresponding *p*-values (*p*). Probability in favor of the Null-Hypothesis *p(H_0_)* has also been included. Samples marked with * were produced by wild wolves. Bold typeface indicates samples where measurements do not follow a Gaussian distribution and have thus been described using robust statistical measurements.

		Mean			Median			Distribution		
Sample 1	Sample 2	*Y*_g_	*p*	*p(H_0_)*	*P*_g_	*p*	*p(H_0_)*	W_g_	*p*	*p(H_0_)*
Wild *	Captive	138.80	0.0001	0.0025	78.53	0.0001	0.0025	69.97	7.3 × 10^−16^	6.9 × 10^−14^
Cabárceno	Flechas *	105.98	0.0001	0.0025	50.94	0.0001	0.0025	47.06	6.0 × 10^−11^	3.8 × 10^−09^
**Cabárceno**	**Hosquillo**	20.98	0.0001	0.0025	16.99	0.0001	0.0025	12.77	1.7 × 10^−03^	2.9 × 10^−02^
Cabárceno	Villardeciervos *	143.00	0.0001	0.0025	55.31	0.0001	0.0025	64.27	1.1 × 10^−14^	9.6 × 10^−13^
Flechas *	**Hosquillo**	50.69	0.0001	0.0025	29.68	0.0001	0.0025	21.69	2.0 × 10^−05^	5.9 × 10^−04^
Flechas *	Villardeciervos *	0.022	0.8864	0.7749	0.22	0.6978	0.5943	4.650	0.1000	0.3850
Hosquillo	Villardeciervos *	59.03	0.0001	0.0025	41.36	0.0001	0.0025	34.08	4.0 × 10^−08^	5.0 × 10^−07^

**Table 3:** Descriptive data for measurements extracted from tooth score cross-sections. Measurements are all reported in mm. Bold typeface indicates samples where measurements do not follow a Gaussian distribution.

Measurement		Cabárceno	Hosquillo	Flechas ^1^	Villardeciervos ^1^	Captive	Wild ^1^
**WIS**	Min.	0.15	0.07	0.11	0.21	0.07	0.11
	Central ^2^	**0.54**	**0.48**	0.47	**0.56**	**0.50**	**0.49**
	Deviation ^3^	**0.40**	**0.27**	0.22	**0.31**	**0.33**	**0.27**
	Max.	1.75	1.78	1.16	1.38	1.78	0.14
**WIM**	Min.	0.11	0.05	0.08	0.15	0.05	0.08
	Central ^2^	**0.37**	**0.33**	**0.29**	**0.39**	**0.34**	**0.33**
	Deviation ^3^	**0.27**	**0.19**	**0.15**	**0.21**	**0.23**	**0.18**
	Max.	1.18	1.20	0.80	0.95	1.20	0.95
**WIB**	Min.	0.06	0.03	0.04	0.08	0.02	0.04
	Central ^2^	**0.19**	**0.17**	**0.16**	**0.21**	**0.18**	**0.17**
	Deviation ^3^	**0.14**	**0.10**	**0.08**	**0.11**	**0.12**	**0.10**
	Max.	0.60	0.61	0.43	0.50	0.61	0.50
**D**	Min.	0.00	0.01	0.02	0.04	0.00	0.02
	Central ^2^	**0.05**	**0.07**	**0.09**	**0.12**	**0.07**	**0.11**
	Deviation ^3^	**0.05**	**0.06**	**0.07**	**0.08**	**0.06**	**0.08**
	Max.	0.24	0.31	0.31	0.37	0.31	0.37
**RDC**	Min.	0.08	0.04	0.06	0.12	0.04	0.06
	Central ^2^	**0.28**	**0.26**	**0.24**	**0.31**	**0.26**	**0.27**
	Deviation ^3^	**0.21**	**0.15**	**0.13**	**0.18**	**0.17**	**0.15**
	Max.	0.90	0.91	0.65	0.76	0.91	0.76
**LDC**	Min.	0.08	0.04	0.06	0.11	0.04	0.06
	Central ^2^	**0.29**	**0.26**	**0.24**	**0.31**	**0.27**	**0.26**
	Deviation ^3^	**0.21**	**0.16**	**0.13**	**0.17**	**0.17**	**0.15**
	Max.	0.90	0.92	0.65	0.78	0.92	0.78

^1^ Wild animal samples. ^2^ Central tendencies are measured as mean or median for Gaussian and non-Gaussian distributed data, respectively. ^3^ Central tendencies are measured as the standard deviation or square root of the biweight mid variance for Gaussian and non-Gaussian distributed data, respectively.

**Table 4:** Absolute difference (|*d*|), *p*-values (*p*), and probability in favor of the null hypothesis (*p(H_0_)*) values obtained from equivalency testing using robust two-one-sided tests on tooth scores.

		Cabárceno	Flechas ^1^	Hosquillo
**Flechas ^1^**	|*d*|	1.779		
	*p*	1.000		
	*p(H_0_)*	0.999		
**Hosquillo**	|*d*|	0.483	1.297	
	*p*	0.689	0.995	
	*p(H_0_)*	0.589	0.986	
**Villardeciervos ^1^**	|*d*|	2.767	0.988	2.285
	*p*	1.000	0.798	1.000
	*p(H_0_)*	1.000	0.671	1.000

^1^ Wild animal samples.

**Table 5:** Absolute difference (|*d*|), *p*-values (*p*), and probability in favor of the null hypothesis (*p(H_0_)*) values obtained from equivalency testing using robust two-one-sided tests on tooth pits.

		Cabárceno	Flechas ^1^	Hosquillo
**Flechas ^1^**	|*d*|	0.062		
	*p*	2.5 × 10^−11^		
	*p(H_0_)*	1.7 × 10^−09^		
**Hosquillo**	|*d*|	0.013	0.050	
	*p*	3.2 × 10^−18^	4.9 × 10^−16^	
	*p(H_0_)*	3.5 × 10^−16^	4.7 × 10^−14^	
**Villardeciervos ^1^**	|*d*|	0.034	0.028	0.021
	*p*	6.8 × 10^−16^	9.4 × 10^−19^	4.4 × 10^−26^
	*p(H_0_)*	6.5 × 10^−14^	1.0 × 10^−15^	7.0 × 10^−24^

^1^ Wild animal samples.

## Data Availability

Data used for the present study have been included as supplementary files.

## Supplementary Materials

The following are available online at https://www.mdpi.com/article/10.3390/ani11082323/s1, Supplementary File S1: Circular Statistics and the Processing of Tooth Score Opening Angles, containing; (1) Definitions of Circular Data, (2) Solutions for Multivariate Analyses. Supplementary File S2: Results for 3D Processing of Tooth Scores. Supplementary File S3: Robust Statistical Measures. Supplementary File S4: Bayesian Modelling. Supplementary File S5: *p*-Value Calibrations. Supplementary File S6: Traditional taphonomic data and variables obtained from the tooth marked samples.
