# Supplementary material for: 3D Insights into the Effects of Captivity on Wolf Mastication and Their Tooth Marks; Implications in Ecological Studies of Both the Past and Present
Source: Animals (Basel). 2021 Aug 6;11(8):2323. doi: 10.3390/ani11082323 (PMC8388415; doi:10.3390/ani11082323)
Supplement: Supplementary file 1 [file animals-11-02323-s001.zip › Supplementary Files S1.pdf]

Effects of Captivity on Carnivore Mastication; Implications in Ecological Studies of both the Past and Present.

Supplementary File S1

Lloyd A. Courtenay[1*], Darío Herranz-Rodrigo[2,3], José Yravedra[2,3], José Mª Vázquez-Rodríguez[4], Rosa Huguet[5,6,7], Isabel Barja[8,9], Miguel Ángel Maté-González[1,10], Maximiliano Fernández Fernández[11,12], Ángel-Luis Muñoz-Nieto[2], and Diego González-Aguilera[1,11]

[1]Department of Cartographic and Terrain Engineering, Higher Polytechnic School of Ávila, University of Salamanca, Hornos Caleros 50, 05003, Ávila, Spain

[2]Department of Prehistory, Complutense University, Prof. Aranguren s/n, 28040, Madrid, Spain

[3]C. A. I. Archaeometry and Archaeological Analysis, Complutense University, Professor Aranguren 2/n, 28040, Madrid, Spain

[4]Department of Prehistory and Archaeology, UNED University. Humanities Faculty, C/ Senda del Rey, 7, 28040, Madrid, Spain

[5]Institut Català de Paleoecologia Humana I Evolució Social (IPHES), Zona Educacional 4, Campus Sescelades URV (Edifici W3), 43700, Tarragona, Spain

[6]Universitat Rovira i Virgili (URV), Department d'Historia I Hostiora de l'Art, Avignuda de Catalunya 35, 43002, Tarragona, Spain

[7]Unit Associated to CSIC, Departamento de Paleobiologia, Museo de Ciencias Naturales, calle José Gutiérrez Abascal, s/n, 28006 Madrid, Spain

[8]Department of Biology, Zoology Unit, Autónoma University of Madrid, C/ Darwin 2, Campus Universitario de Cantoblanco, 28049, Madrid, Spain

[9]Center of Investigation in Biodiversity and Global Change (CIBC-UAM), Universidad Autónoma de Madrid, Madrid, Spain.

[10]Department of Topographic and Cartography Engineering, Higher Technical School of Engineers in Topography, Geodesy and Cartography, Technical University of Madrid, Mercator 2, 28031, Madrid, Spain

[11]Gran Duque de Alba Institution, Dibutación Provincial de Ávila, Paseo Dos de Mayo, 8, 05001, Ávila, Spain

[12]Department of Sciences of Communication and Sociology, Faculty of Communication Sciences, University Rey Juan Carlos, Camino del Molino, s/n, 28943, Fuenlabrada, Madrid, Spain

[*]Corresponding author. Email: ladc1995@gmail.com

August 6, 2021

Contents

1 Introduction **2**

2 Problem Presentation **2**

3 Definitions for Circular Data **4**

3.1 Calculating Central Tendency 5

3.2 Summary Statistics for Sample Concentration and Dispersion 7

3.3 Comparing Samples and Null Hypothesis Testing 10

4 Solutions for Multivariate Analyses **11**

4.1 Autoencoder Methods 12

4.2 Multivariate Results 14

1 Introduction

The present document contains the supplementary materials for the article titled **Effects of Captivity on Carnivore Mastication; Implications in Ecological Studies of both Past and Present**. Here we list the analytical procedures employed for the analysis of the variable "Opening Angle" (OA), including the mathematical equations used to define concepts within the text of our paper.

The original definition of this variable in the metric study of taphonomic traces was proposed by Silvia Bello and Christophe Soligo [1], calculated via the angle between the slope S_1 and S_2, thus defining the Opening Angle (OA), or δ (Fig. S1.1). These values are reported in degrees (°), and have typically been processed using traditional univariate statistical methods [1], or in combination with other metric variables via Principal Components Analyses and other multivariate approaches [2, 3]. In recent years, however, studies have noted on a number of occasions how the variable OA has often held a unique weight on statistical tests [4, 5], in some cases producing noise. Few attempts, however, have been made to try and understand this statistical noise and evaluate the most efficient means of integrating this data. The present supplementary file presents an update to the already present analytical procedures in order to improve the processing of such data.

2 Problem Presentation

Circular data, in comparison with linear data, can be described to have a number of differential components. For simplicity the present document will note 3 of these; location of the origin, differences in calculation of mean, and finally the distortion produced by combining these factors when projected onto a measurable scale.

- The origin of a linear scale is simply defined as 0. Under this premise any value to the left of 0 are noted as negative values, while values to the right are positive. Considering this, the value

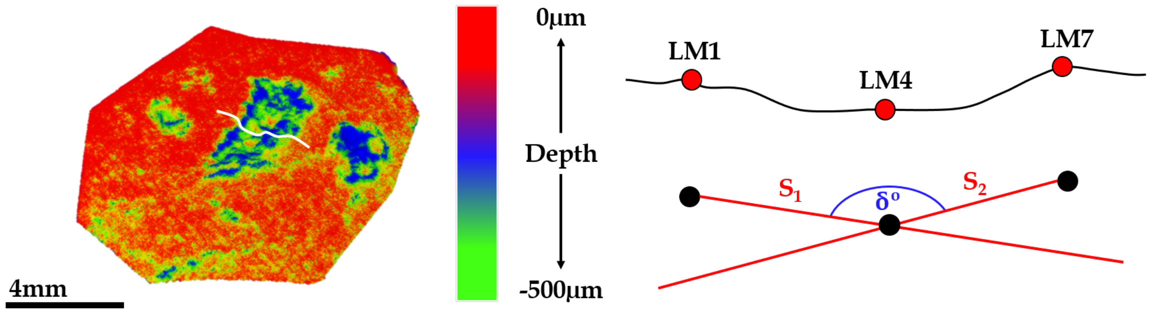

Supplementary Figure S1. 1: *Obtaining measurements of opening angle for taphonomic traces. Left: 3D elevation model in Cloud Compare. Right: cross section of the mark under staudy, the landmarks used to derive measurements for OA and the definition of δ by [1] and [5].*

355 is considered to be close to 360, yet far away from 0. When considering angles measured in degrees, however, this is not always the case. On a circular scale, the value 360° is often synonymous with the value 0°, therefore (1) values to the left of 0 do not necessarily have to be negative values, while (2) 355° is just as close to 0° as it is 360°. Furthermore, in many practical cases the measurement 370° is not possible while the linear value 370 is completely plausible.

- Linear means and circular means vary, and in some cases greatly. Consider the values 5, 10, 350 and 355. The arithmetic mean of these 4 values is defined as (eq. 1):

$$\bar{x} = \frac{1}{n}\sum_{i=1}^{n} x_i \tag{1}$$

However, if we were to consider these values as directions 5°, 10°, 350° and 355°, the arithmetic mean calculated via this formula (eq. 2) consequently equates to 180°.

$$\therefore \frac{350 + 355 + 5 + 10}{4} = 180 \tag{2}$$

This value evidently points in the complete opposite direction and therefore is not a valid representation of these numbers (Fig. S1.2).

- Taking into consideration the aforementioned points, the projection of circular data onto a linear plane can consequently result in large amounts of distortion. The easiest means of explaining this concept is to consider coordinate projections in geodesy (Fig. S1.3). Depending on our choice of projection, measurements can vary greatly, and in many cases traditional calculations for euclidean distances and arithmetic means cease to remain reliable. Under this premise the majority of statistical tests loose credibility, as null-hypotheses in parametric statistics are often derived from distribution means.

Upon evaluating these three components, the authors believe care must be taken when defining the variable OA and its underlying properties. Traditional statistical tests, therefore, can be con-

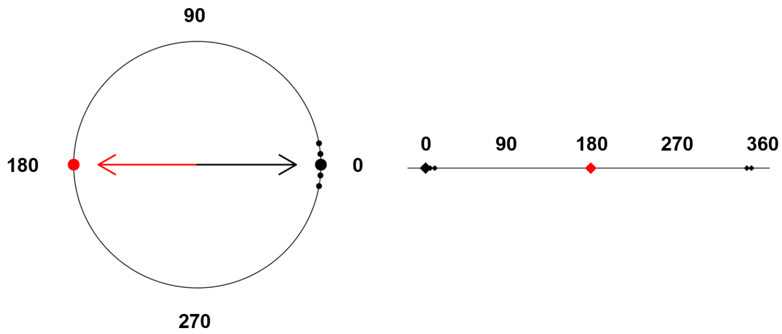

Supplementary Figure S1. 2: *Example of the circular (black) and linear (red) mean for values 5, 10, 350 and 355.*

sidered ineffective when processing this information, while the variable's redefinition is fundamental before it can be successfully integrated into future multivariate statistical analyses.

3 Definitions for Circular Data

Considering the trigonometric properties of circular data, traditional statistics usually used on typical *linear* data are likely to create distortion when applied to angles. This thus requires a more specified branch of statistics in order to extract precise empirical inferences about the dataset. Here we will use the term *circular statistics* to make reference to such analyses.

The first condition to consider when processing circular data for angles are the units; degrees (x°) or radians (rad or x^c). The measurements performed in the present study were all extracted and measured in degrees. Nevertheless, for ease of mathematical processing, these values have all been converted into radians (eq. 3). All values reported in tables within the main manuscript have been converted back to degrees (eq. 4). The basic conversion from degrees to radians can simply be considered as:

$$x^c = x^\circ \frac{\pi}{180} \tag{3}$$

$$x^\circ = x^c \frac{180}{\pi} \tag{4}$$

Likewise, angles can also be represented in accordance with their position to the origin, with positive values usually indicating angles measured in a clockwise direction from the source. Regardless of the units, however, a simple angle is typically represented in literature by θ, while the present study will make use of only absolute values, or $|\theta|$ [1].

[1] Please note that authors Bello and Soligo originally defined this variable using the symbol δ. In continuation this document will refer to δ as θ, as is most common in geometric literature

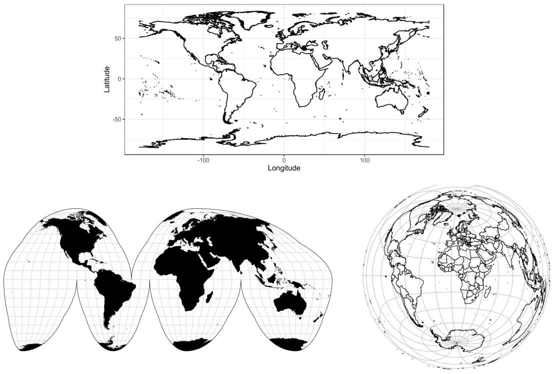

Supplementary Figure S1. 3: *Examples of distortion induced by linear projections of circular data. Top panel: World Geodetic System 1984 Projection (Map created using base R plot functions and the rgdal library). Bottom left: Goode Homolosine Projection (Map created using the d3-geo-projection Javascript library). Bottom Right: Lambert Azimuthal Equal-Area Projection (Map created using base R plot functions and the rgdal library).*

Representations of angles in general can be defined as unit vectors, $\vec{v}$, describing a point directed from the center of a circle to its corresponding position on the circle itself (Fig. S1.4a). This information, as with any vector in linear algebra, is represented by both magnitude and direction. In the case of a single observation, we can define the point z in Figure S1.4 as an observation $\approx 150°$ or $\approx 2.6^c$ clockwise from the origin (0). $\vec{v}$ thus defines the direction from the center of the circle to point z.

The position of this point can be additionally projected onto a planar coordinate system (Fig. S1.4b), using the trigonometric function (eq. 5):

$$(x, y) = (\vec{v}\, cos\theta, \vec{v}\, sin\theta) \tag{5}$$

Considering, however, that the objective of our study is to understand the location of a set of observations, the magnitude of the vector ($\vec{v}$) can be equated to 1, thus simplifying our formula to the following equation (eq. 6):

$$(x, y) = (cos\theta, sin\theta) \tag{6}$$

3.1 Calculating Central Tendency

When working with a set of observations of length n, vectors $\vec{x}_1, ..., \vec{x}_n$ consisting of angles $\theta_1, ..., \theta_n$ and complex numbers $z_1, ..., z_n$ can be summarised using their corresponding *Trigonometric Moments*. In the simplest terms, a sample trigonometric moment is defined through $a_p + ib_p$, where p refers to the p_{th} moment around the zero direction. a_p and b_p are defined through (eq. 7):

$$a_p = \frac{1}{n}\sum_{j=1}^{n} cos\, p\theta_j\ ,\ b_p = \frac{1}{n}\sum_{j=1}^{n} sin\, p\theta_j \tag{7}$$

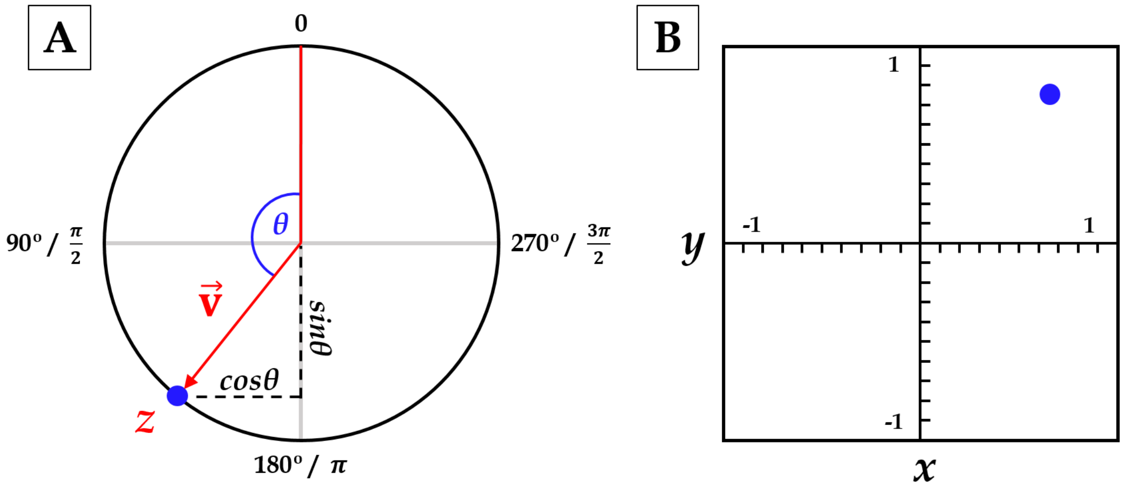

Supplementary Figure S1. 4: *Example of a single measured angle represented in (A) circular space as well as in (B) linear space*

These values can be used to define the mean resultant vector's length (eq. 8) and direction (eq. 9 & 10) on a complex plane:

$$\bar{R}_p = \left(a_p^2 + b_p^2\right)^{1/2} \in [0, 1] \tag{8}$$

$$\bar{\theta}_p = atan2\,(b_p, a_p) \tag{9}$$

$$\text{atan2}\,(b_p, a_p) = \begin{cases} \arctan(b_p/a_p), & a_p > 0, \\ \arctan(b_p/a_p) + \pi, & b_p \geq 0, \\ \arctan(b_p/a_p) - \pi, & b_p < 0, \\ \pi/2, & b_p > 0, a_p = 0, \\ -\pi/2, & b_p < 0, a_p = 0, \\ \text{undefined}, & b_p = 0, a_p = 0, \end{cases} \tag{10}$$

The sample mean direction ($\bar{\theta}$) can therefore be calculated through (eq. 11 to 13):

$$Y = \frac{\sum_{i=1}^{n} sin_\theta}{n}\ ,\ X = \frac{\sum_{i=1}^{n} cos_\theta}{n} \tag{11}$$

$$r = \sqrt{X^2 + Y^2} \tag{12}$$

$$cos\ \bar{a} = \frac{X}{r},\ sin\ \bar{a} = \frac{Y}{r},\ \bar{\theta} = \arctan\left(\frac{sin\ \bar{a}}{cos\ \bar{a}}\right) \tag{13}$$

and sample mean resultant length ($\bar{R}$, eq. 14):

$$\bar{R} = \frac{1}{n}\sum_{j=1}^{n} cos\left(\theta_j - \bar{\theta}\right) \quad (14)$$

Sample medians ($\tilde{\theta}$) are calculated as any angle ψ that lies on the midway point of an arc $[\psi, \psi + \pi)$, with median direction (eq. 15):

$$\frac{1}{n}\sum_{j=1}^{n}\{\pi - |\pi - |\theta_j - \psi||\} \quad (15)$$

3.2 Summary Statistics for Sample Concentration and Dispersion

The trigonometric moments provide a number of useful measurements that can be used to define the nature of a set of observations. The second trigonometric moment ($p = 2$) is where the majority of information regarding skewness and kurtosis can be obtained. We can define the second central sine (eq. 16) and cosine (eq. 17) moments through:

$$\bar{b}_2 = \frac{1}{n}\sum_{j=1}^{n} sin\,2\left(\theta_j - th\bar{e}ta\right) \quad (16)$$

$$\bar{a}_2 = \frac{1}{n}\sum_{j=1}^{n} cos\,2\left(\theta_j - th\bar{e}ta\right) \quad (17)$$

Which can additionally be converted into standardised measures [6]:

$$\hat{s} = \frac{\bar{b}_2}{\left(1 - \bar{R}\right)^{3/2}} \quad (18)$$

$$\hat{k} = \frac{\bar{a}_2 - \bar{R}^4}{\left(1 - \bar{R}\right)^{2}} \quad (19)$$

for skewness (eq. 18) and kurtosis (eq. 19), respectively.

An additional metric used in circular statistics is the sample circular variance (V), which is simply measured via $V = 1 - \bar{R}$, where $V \in [0, 1]$ with 1 indicating high concentrations of observations across the circle.

From each of the aforementioned summary statistics, a number of conclusions can be withdrawn regarding the normality of our data. Nevertheless, in order to decide whether parametric or non-parametric tests should be used for further inference, two additional components must be considered; *uniformity* and *symmetry*.

Uniformity is tested considering the measurements of concentration previously defined through $\bar{R}$ (eq. 14). The null-hypothesis in this case considers data to be uniformly distributed around a circle when $\bar{R}$ is no greater than a defined threshold. The best means of testing for this is via the Rayleigh test [7].

Depending on the type of data being tested, however, uniformity is not always a precise means of testing for deviations from normality. When considering how certain observations are not likely to

appear across the entire spectrum of the circular scale, uniformity is, more often than not, absent. Under this premise, symmetry is a more empirical means of testing for statistical corruption in circular data.

Symmetry is measured considering the distribution of $\bar{b}_2$ (eq. 16 & 18) around $\bar{\theta}$. The presence of reflective symmetry can be assessed using the following test statistic (eq. 20 & 21):

$$z = \frac{\bar{b}_2}{\sqrt{v\hat{a}r\left(\bar{b}_2\right)}} \tag{20}$$

$$v\hat{a}r\left(\bar{b}_2\right) = \frac{1}{n}\left[\frac{1-\bar{a}_4}{2} - 2\bar{a}_2 + \frac{2\bar{a}_2}{\bar{R}}\left\{\bar{a}_3 + \frac{\bar{a}_2\left(1-\bar{a}_2\right)}{\bar{R}}\right\}\right] \tag{21}$$

where large values of z lead to rejection of the null hypothesis, indicating a notably skewed distribution [8]. A robust extension of this test can then be obtained through employing traditional bootstrap procedures in statistics [9]. p-values can then additionally be assigned confidence intervals through (eq. 22):

$$\hat{p} \pm z_{(1-\alpha/2)}\sqrt{\frac{\hat{p}\left(1-\hat{p}\right)}{\left(N_R+1\right)}} \tag{22}$$

where N_R are the number of permutations or repeats, typically set to 10,000 [8, 10].

Finally, a note has to be made on the definition of “normality” in circular data. As opposed to linear statistics, where a Gaussian distribution describes a bell-shaped curve, inferring normality on circular statistics can often be complicated and will be dependent on the domain being studied. Under this premise, a number of distribution types have been defined [11–15] (Fig. S1.5). Much can be said on the properties and definitions of each distribution type, however this goes far beyond the scope of the present document.

After careful analysis of each of the proposed representations, the variable OA can be considered closest to the *Von Mises Distribution* [13–15] (Fig. S1.6 & Table S1.1).

Supplementary Table S1. 1: *Statistical Comparison between different hypothetical distributions and the measurements OA. Test statistic used corresponds to the Mardia-Watson-Wheeler test (consult section "Comparing Samples and Null Hypothesis Testing"). $p\left(H_0\right)$ have also been included.*

Hypothetical Distribution	W_g	p-value	$p\left(H_0\right)$
Cardioid	185.76	4.6e-41	1.2e-38
Wrapped Cauchy	114.59	1.3e-25	2.0e-23
Wrapped Normal	3.53	0.17	0.45
von Mises	0.07	0.25	0.49

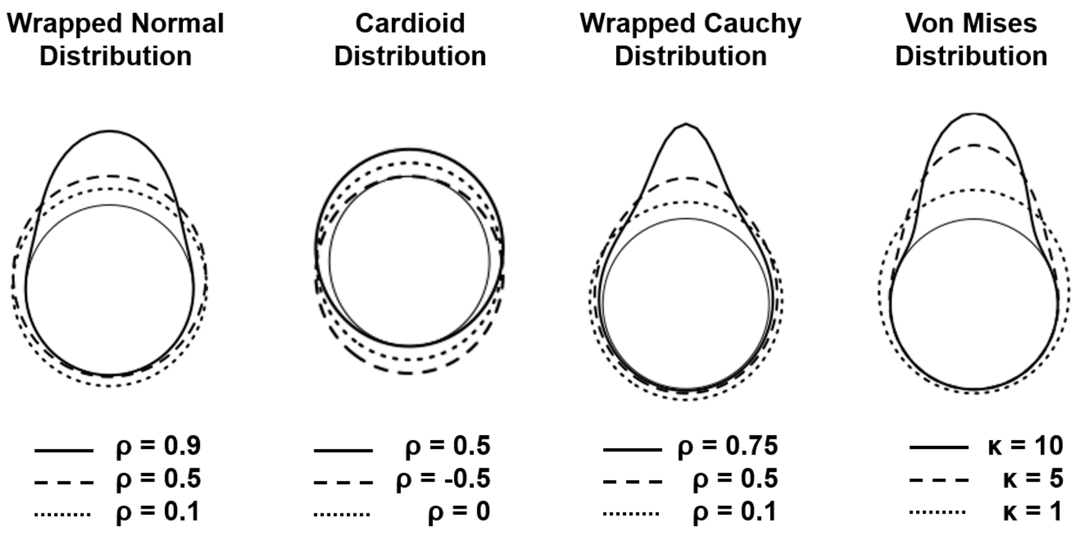

Supplementary Figure S1. 5: *Examples of 4 useful circular distribution types; the Wrapped Normal [13], Cardioid [11], Wrapped Cauchy [12] and Von Mises [13–15]*

The von Mises distribution is described to have density (eq. 23):

$$f(\theta) = \frac{e^{\kappa\ cos(\theta-\mu)}}{2\pi I_0(\kappa)} \tag{23}$$

where μ defines the mean direction and κ is defined as the concentration parameter (eq. 24):

$$I_p(\kappa) = \frac{1}{2\pi}\int_0^{2\pi} cos\ p\theta e^{\kappa\ cos\theta} d\theta \tag{24}$$

The *Von Mises Distribution* usefully describes a series of observations that are concentrated to one particular area of the overall "circular scale". In this sense the density function (eq. 24) best suits the variable OA considering how values of 10° or 270° are unlikely to occur in a cut mark

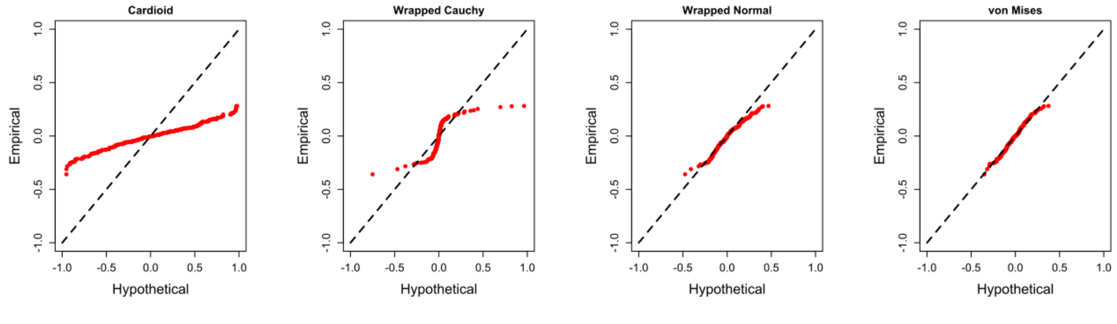

Supplementary Figure S1. 6: *Percentile-Percentile plots comparing OA measurements with 4 theoretical distributions; the Wrapped Normal [13], Cardioid [11], Wrapped Cauchy [12] and Von Mises [13–15]*

or tooth score dataset. This function, therefore, resembles the "bellcurve" component of a linear Gaussian distribution, yet is conveniently wrapped in a circular feature space.

Hypothesis testing for a goodness-of-fit comparing our data with the Von Mises Distribution is best performed using a *Maximum Likelihood Estimation* with bias correction [16]. Goodness-of-fit tests can be performed visually using Quantile-Quantile and Probability-Probability plots, or quantitatively via a direct comparison between a theoretically constructed Von Mises Distribution and the target distribution. More notes on this can be consulted in the "Comparing Samples and Null Hypothesis Testing" section of the present document. Results can be bootstrapped to construct both confidence intervals and more robust test-statistics via equation 22.

Finally, distributions can be visualised through simple scatter plots and enhanced via a *kernel density* function. Kernel's only require the definition of a single parameter, known as the *bandwidth*. This can be empirically defined using cross-validation to minimize the error of the correspondent representation. The present study uses Mean Squared Error loss functions for kernel bandwidth estimation [17].

3.3 Comparing Samples and Null Hypothesis Testing

Combining all of the above, and in accordance with standard practice in both traditional and robust statistics, comparing samples can be performed considering differences in either mean or median for normally distributed and non-normally distributed observations respectively. Furthermore, spherical data can additionally be studied using two additional tests. These tests are used to describe concentration and overall distribution properties. Considering how concentrations are not likely to vary greatly within our datasets, the present study focuses solely on differences in distribution.

Hypothesis Testing for differences in Mean

A common statistical test for the evaluation of differences between two or more means was originally proposed by Watson [18], with a bootstrap alternative for more robust calculations avoiding issues related to sample size [16, 19]. The test statistic, Y_g, used for this test is dependent on the circular dispersion of the samples (g). For this Watson defined two separate statistics P (eq. 25 & 27) and M (eq. 26 & 28);

$$Y_g^P = \frac{2\left(N - R_p\right)}{\hat{\delta}_0} \tag{25}$$

$$Y_g^M = 2\left(\sum_{k=1}^{g} \frac{n_k}{\hat{\delta}_k} - R_M\right) \tag{26}$$

$$R_P = \sqrt{\hat{C}_P^2 + \hat{S}_P^2}, \;\; \hat{C}_P^2 = \sum_{k=1}^{g} n_k \, cos \, \bar{\theta}_k, \;\; \hat{S}_P^2 = \sum_{k=1}^{g} n_k \, sin \, \bar{\theta}_k, \;\; \hat{\delta}_0 = \sum_{k=1}^{g} n_k \frac{n_k \hat{\delta}_k}{N} \tag{27}$$

$$R_M = \sqrt{\hat{C}_M^2 + \hat{S}_M^2}, \;\; \hat{C}_M^2 = \sum_{k=1}^{g} \frac{n_k \, cos \, \bar{\theta}_k}{\hat{\delta}_k}, \;\; \hat{S}_M^2 = \sum_{k=1}^{g} \frac{n_k \, sin \, \bar{\theta}_k}{\hat{\delta}_k} \tag{28}$$

Choice of M or P considers the ratio of the largest $\hat{\delta}_g$ values divided by the smallest. If the resultant value is less than 4, then the P procedure is used [16, 19, 20].

Hypothesis Testing for differences in Median

Watson's Y_g test is generally considered non-parametric as it does not make any underlying assumptions on distribution dispersion or shape. Nevertheless, the bootstrapped versions proposed by Fisher and Hall [16, 19] require slight adjustments according to underlying symmetry among the distributions. Under this premise, for samples with large $\hat{s}$ and z values (eq. 18 & 20), alternatives should be considered using sample medians.

Fisher's non-parametric proposal [16] consists in first calculating the median direction ($\tilde{\theta}$) for all points in the combined samples. For each of the samples, k, m_k is used to denote the number of negative values resultant of $\theta_{k1} - \tilde{\theta}$, ..., $\theta_{kn_k} - \tilde{\theta}$. Additionally, each of the $\theta_{kj} - \tilde{\theta}$, $j = 1$, ..., n_k are calculated to lie $\in (-\pi, \pi]$, and M is thus all the consequent negative values. The null-hypothesis can then be defined through the test statistics P_g where excessively large values reject the possibility that distributions g share a common median (eq. 29).

$$P_g = \frac{N^2}{M(N-M)} \sum_{k=1}^{g} \frac{m_k^2}{n_k} - \frac{NM}{N-M} \tag{29}$$

For robustness, randomization is a commonly used permutation procedure and strongly recommended [16, 19, 20].

Hypothesis Testing for differences in Distribution

A popular test for assessing differences in distributions is the Mardia-Watson-Wheeler test [6, 21]. This rank based test first groups all measurements into a single vector, ranking them using any random direction. From here R_{kj} is defined as the rank of element j in sample k, which can be plugged in to calculate the test statistic through (eq. 30 & 31):

$$W_g = s \sum_{k=1}^{g} \frac{C_k^2 + S_k^2}{n_k} \tag{30}$$

$$C_k = \sum_{j=1}^{n_k} cos\left(\frac{2\pi R_{kj}}{N}\right),\ S_k = \sum_{j=1}^{n_k} sin\left(\frac{2\pi R_{kj}}{N}\right) \tag{31}$$

If the test statistic is high, then the difference between distributions can be considered important. As with the previous example, for robust calculations regardless of sample size, randomization permutations are recommended [16, 19, 20].

4 Solutions for Multivariate Analyses

In light of the definitions presented within this document, and in order to find the best means of incorporating circular data into multivariate statistical analyses combined with linear variables,

multiple experiments were performed using autoencoders for Principal Components Analysis (PCA). An autoencoder is a neural network consisting of two parts; (1) the encoder and (2) the decoder [22, 23]. These networks are trained to produce an output which is a reconstructed copy of the input. Autoencoders are usually designed using an hourglass shape with smaller hidden layers in the middle so as to reproduce the effect of compression and dimensionality reduction on the input (Fig. S1.7). The Encoder is thus used to literally *encode* the inputs, representing said inputs as a compressed vector, while the decoder reconstructs the input from this compressed representation. The output from the decoder can then be compared with the input so as to assess the quality of the encoded representation. These techniques have traditionally been known to be particularly useful for dimensionality reduction and feature learning [23–25].

4.1 Autoencoder Methods

The advantage of using autoencoders for PCA lies in the ability to benchmark and assess dimensionality reduction performance via measurable metrics. For the purpose of this study, Mean Squared Error (MSE, eq. 32) was chosen.

$$MSE = \frac{1}{N}\sum_{i=1}^{N}(y_i - \hat{y}_i)^2 \tag{32}$$

To recreate the functionality of PCA via a Neural-Network based architecture, the autoencoder was programmed to include a single hidden layer containing the reduced dimensions of the data (Fig. S1.7). The size of this hidden layer was fixed at 2 considering how traditional PCA of the respected metric variables usually represents >99% of the total variance. To further replicate the underlying parametric components of PCA, only linear activation functions were used. Following this, 4 additional properties had to be established in order to ensure the autoencoder has the same properties as PCA. These include;

1. Tied weights between the encoder and the decoder
2. Orthogonal weights to ensure each vector is independent from the others
3. Uncorrelated features to ensure that outputs are uncorrelated
4. "Unit Norm" across all weights

Linked with property 1, no bias was used on either the encoder or the decoder to ensure that reconstruction error was entirely dependent on the variables rather than the configuration of the model.

The final Principal Component Autoencoder (PC-Autoencoder) was then trained for 100 epochs with batch sizes of 8. Weights were optimized using a standard Stochastic Gradient Descent algorithm with the default learning rate. MSE (eq. 32) was used as the loss function. All variables were additionally scaled prior to training. For each of the trials, the variable OA was input into the model using different formats to assess which of the representations of OA were able to produce the least amount of reconstruciton error (Fig. S1.7). These representations include (Fig. S1.8):

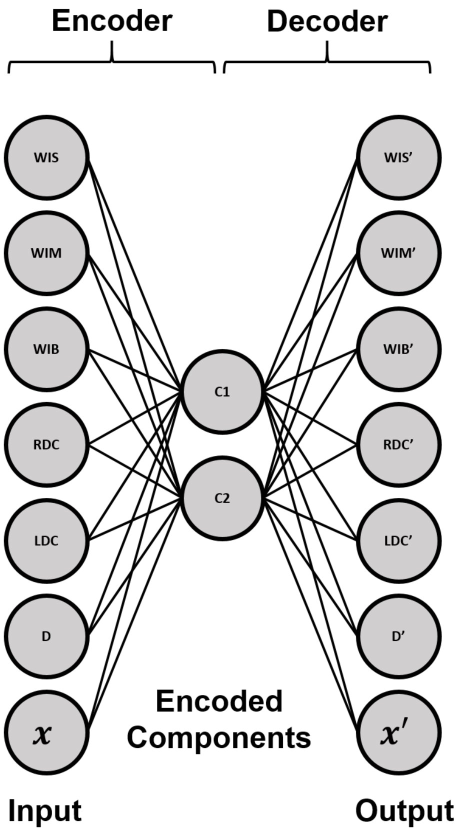

Supplementary Figure S1. 7: *Autoencoder architecture for Principal Component Analysis (PC-Autoencoder). Node with variable x varies according to the representation of OA being used. Please note that the bias neuron has been removed from this visualisation for simplicity.*

1. OA recorded in *degrees* (θ°)
2. OA recorded in *radians* (θ^{c})
3. Two separate neurons including $cos\theta$ and $sin\theta$ values (x & y).
4. A single neuron including a combination of linearly transformed θ values, defined through $cos\theta + sin\theta$ $(x + y)$.

Finally, considering how weight initialisation is a stochastic process, training was permuted 30 times per trial and summary statistics were used to report the final results.

Alongisde reconstruction error analyses, tests were performed to observe element loadings, considering the weight of each variable (x_k) that is extracted through the corresponding eigenvector ($\vec{e}_m$) for each of the individual elements $e_{k,m}$.

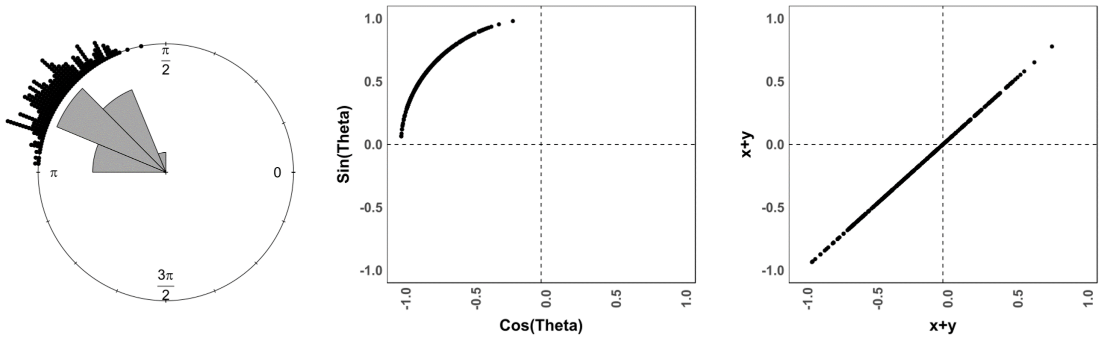

Supplementary Figure S1. 8: *Opening Angles from the present study converted into (x,y) coordinates and then combined as a single linear component*

4.2 Multivariate Results

PC-Autoencoders revealed the optimal method for incorporating opening angles into multivariate statistics is through the combination of linearly transformed radians; $cos\theta + sin\theta$ or $x + y$ (Fig. S1.9, Table S1.2).

It can clearly be seen through the results presented here that attempting to employ parametric linear functions to circular data produces a notable increase in multivariate error. Likewise, results of matrix dimensionality reduction including θ° was less consistent than in all other cases (Deviation values in Table S1.2). Finally it can be seen how combining the linearly transformed circular variables into a single values improves the quality of dimensionality reduction (Kruskal-Wallis Chi^2, $p = 0.025$), thus demonstrating that $cos\theta + sin\theta$ is the most reliable means of incorporating circular data into multivariate analyses.

When considering the effects of variable transformations on each of the $\vec{e}_m$, interestingly, the magnitude of OA-θ° is substantially larger than any of the other variables. While the effects of scaling or conversion of OA-θ° into OA-θ^c is likely to correct this, the weight OA-θ° has on the overall variation in feature space is a factor worth mentioning.

Finally, considering correlation plots (Fig. S1.9) reveal very high correlations between WIS, WIM, WIB, RDC and LDC variables, it may be argued that the real issue in these multivariate analyses is the overly correlated nature of these variables. This is a factor that is likely to mask the true influence of other variables, especially in the case of tooth scores, possibly indicating that variables such as WIM and WIB are redundant when paired with WIS.

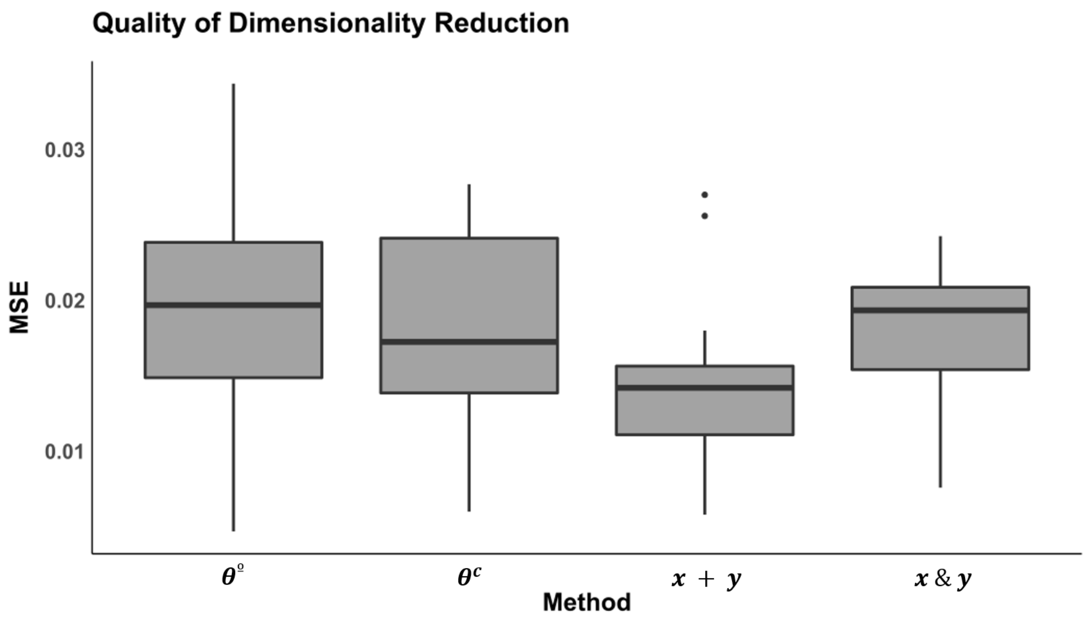

Supplementary Figure S1. 9: *Boxplots of Mean Squared Error (MSE) values obtained from 30 training iterations of the PC-Autoencoder using different representations of the variable OA*

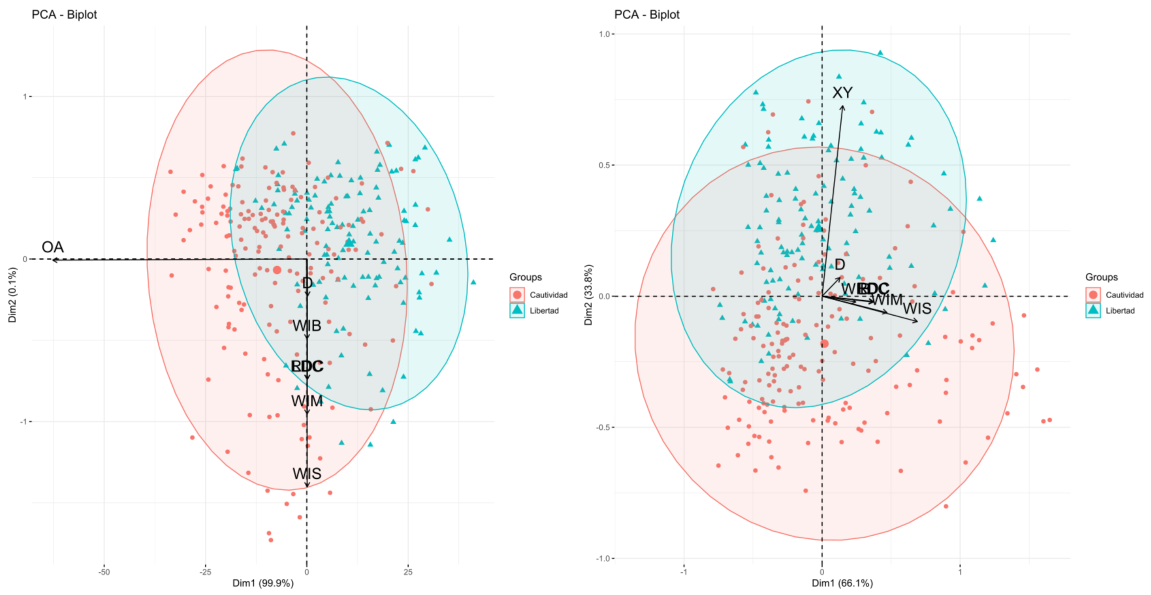

Supplementary Figure S1. 10: *Principal Component Analysis Biplot with Variable Loadings. Left panel including OA in degrees. Right panel including OA as linearly transformed $(x + y)$ variables. IMPORTANT NOTE: For ease of visual representations plot axes do not have equal scales*

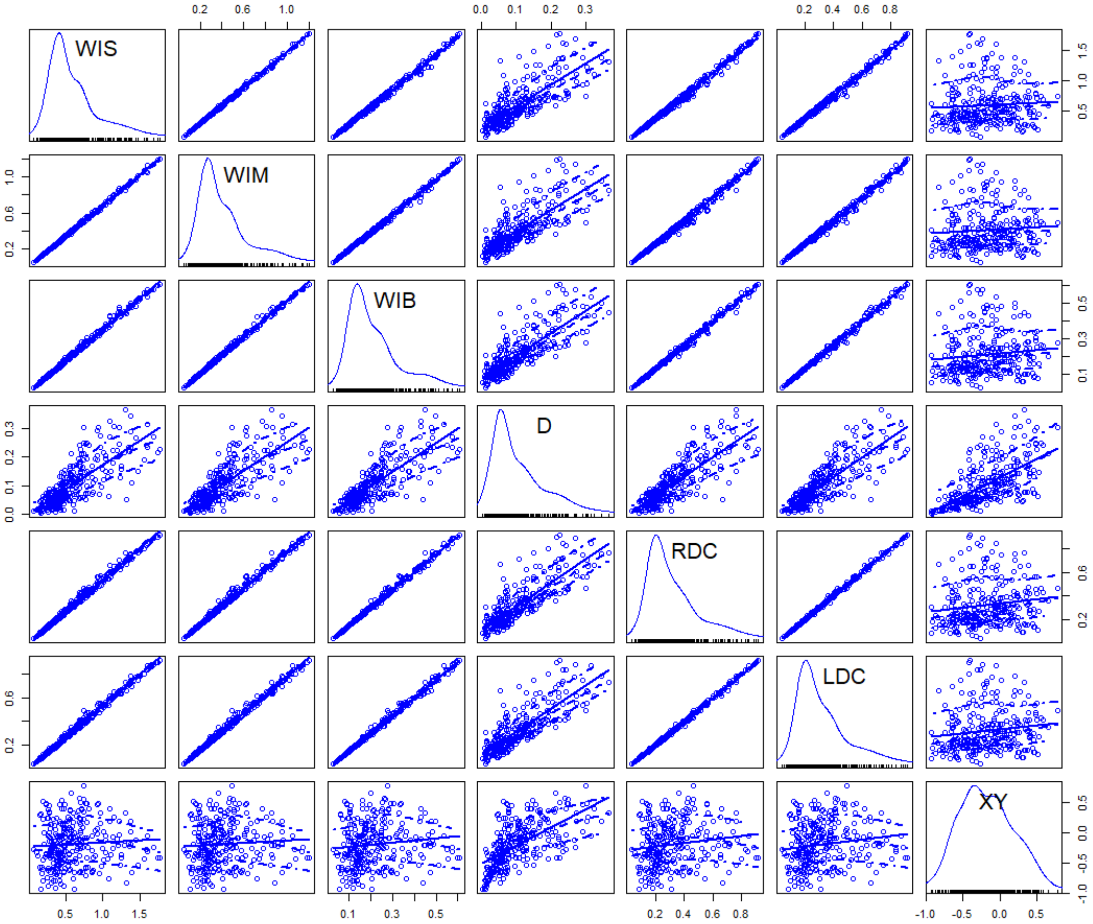

Supplementary Figure S1. 11: *Scatter plot matrix for all variables*

Supplementary Table S1. 2: *Descriptive statistics for Mean Squared Error (MSE) values obtained from 30 training iterations of the PC-Autoencoder using different representations of the variabel OA*

Measurement	Degrees	Radian	x + y	x & y
Shapiro w	0.97	0.95	0.897	0.91
Shapiro p	0.63	0.17	0.007	0.02
IR 0.05 Limit	0.005	0.006	0.06	0.008
Central Tendency*	0.019	0.018	0.014	0.019
Deviation†	0.007	0.007	1.6e-05	0.008
IR 0.95 Limit	0.032	0.028	0.026	0.023

* Variations of the central tendency metric include the mean for Gaussian distributions and the median for non-Gaussian distributions; † Variations of the deviation metric include the standard deviation for Gaussian distributions and the Biweight Midvariance for non-Gaussian distributions

References

[1] S. Bello and C. Soligo, "A new method for the quantitative analysis of cutmark micromorphology," *Journal of Archaeological Science*, vol. 35, pp. 1542–1552, 2008. DOI: 10.1016/j.jas.2007.10.018.

[2] M. Á. Maté-González, J. Yravedra, D. González-Aguilera, J. F. Palomeque-González, and M. Domínguez-Rodrigo, "Micro-photogrammetric characterization of cut marks on bones," *Journal of Archaeological Science*, vol. 62, pp. 128–142, 2015. DOI: 10.1016/j.jas.2015.08.006.

[3] J. Yravedra, E. G. Vargas, M. Á. Maté-González, J. Aramendi, J. Palomeque-González, J. Vallés-Iriso, J. Matesanz-Vicente, D. González-Aguilera, and M. Domínguez-Rodrigo, "The use of micro-photogrammetry and geometric morphometrics for identifying carnivore agency in bone assemblage," *Journal of Archaeological Science: Reports*, vol. 14, pp. 106–115, 2017. DOI: 10.1016/j.jasrep.2017.05.043.

[4] L. A. Courtenay, M. Á. Maté-González, J. Aramendi, J. Yravedra, D. González-Aguilera, and M. Domínguez-Rodrigo, "Testing accuracy in 2D and 3D geometric morphometric methods for cut mark identification and classification," *PeerJ*, 2018. DOI: 10.7717/peerj.5133.

[5] M. Á. Maté-González, J. Aramendi, J. Yravedra, R. Blasco, J. Rosell, D. González-Aguilera, and M. Domínguez-Rodrigo, "Assessment of statistical agreement of three techniques for the study of cut marks: 3d digital microscope, laser scanning confocal microscopy and micro-photogrammetry," *Journal of Microscopy*, vol. 267(3), pp. 356–370, 2017. DOI: 10.1111/jmi.12575.

[6] K. V. Mardia, *Statistics of Directional Data.* London: Academic Press, 1972.

[7] G. S. Watson and E. J. Williams, "On the construction of significance tests on the circle and on the sphere," *Biometrika*, vol. 43, pp. 344–352, 1956.

[8] A. Pewsey, "Testing circular symmetry," *Canadian Journal of Statistics*, vol. 30, pp. 591–600, 2002.

[9] B. Efron, "Bootstrap methods: Another look at the jackknife," *Annals of Statistics*, vol. 7, pp. 1–26, 1979.

[10] B. F. J. Manly, *Randomisation, Bootstrap and Monte Carlo Methods in Biology.* Boca Raton: Chapman and Hall, 2007.

[11] H. Jeffreys, *Theory of Probability.* Oxford: Oxford University Press, 1948.

[12] P. McCullagh, "Möobius transformation and cauchy parameter estimation," *Annals of Statistics*, vol. 24, pp. 787–808, 1996.

[13] K. V. Mardia and P. E. Jupp, *Directional Statistics.* Chichester: John Wiley, 1999.

[14] D. Best and N. Fisher, "Efficient simulation of the von mises distribution," *Applied Statistics*, vol. 28, pp. 152–157, 1979.

[15] R. von Mises, "Über die "ganzzahligkeit" der atomgewichte und verwandte fragen," *Physikalische Zeitschrift*, vol. 19, pp. 490–500, 1918.

[16] N. I. Fisher, *Statistical Analysis of Circular Data.* Cambridge: Cambridge University Press, 1993.

[17] P. Hall, G. S. Watson, and J. Cabrera, "Kernel density estimation with spherical data," *Biometrika*, vol. 74, pp. 751–762, 1987.

[18] G. S. Watson, *Statistics on Spheres.* New York: John Wiley, 1983.

[19] N. I. Fisher and P. G. Hall, "Bootstrap algorithms for small samples," *Journal of Statistical Planning and Inference*, vol. 27, pp. 157–169, 1991.

[20] A. Pewsey, M. Neuhäuser, and G. D. Ruxton, *Circular Statistics in R.* Oxford: Oxford University Press, 2013.

[21] S. Wheeler and G. S. Watson, "A distribution-free two-sample test on the circle," *Biometrika*, vol. 51, pp. 256–257, 1964.

[22] I. Goodfellow, Y. Bengio, and A. Courville, *Deep Learning.* Cambridge: MIT Press, 2016.

[23] H. Bourland and Y. Kamp, "Auto-association by multilayer perceptrons and singular value decomposition," *Biological Cybernetics*, vol. 59, pp. 291–294, 1988.

[24] G. E. Hinton and R. S. Zemel, "Autoencoders, minimum description length, and helmholtz free energy," in *NIPS*, 1994.

[25] V. N. Marivate, F. V. Nelwamondo, and T. Marwala, "Autoencoder, principal component analysis and support vector regression for data imputation," *CoRR*, 2007. arXiv: `0709.2506`.
