# Supplementary material for: 3D Insights into the Effects of Captivity on Wolf Mastication and Their Tooth Marks; Implications in Ecological Studies of Both the Past and Present
Source: Animals (Basel). 2021 Aug 6;11(8):2323. doi: 10.3390/ani11082323 (PMC8388415; doi:10.3390/ani11082323)
Supplement: Supplementary file 1 [file animals-11-02323-s001.zip › Supplementary Files S2 to S6.pdf]

Effects of Captivity on Carnivore Mastication; Implications in Ecological Studies of both the Past and Present.

Supplementary Files S2 to S6

Lloyd A. Courtenay[1*], Darío Herranz-Rodrigo[2,3], José Yravedra[2,3], José Mª Vázquez-Rodríguez[4], Rosa Huguet[5,6,7], Isabel Barja[8,9], Miguel Ángel Maté-González[1,10], Maximiliano Fernández Fernández[11,12], Ángel-Luis Muñoz-Nieto[2], and Diego González-Aguilera[1,11]

[1]Department of Cartographic and Terrain Engineering, Higher Polytechnic School of Ávila, University of Salamanca, Hornos Caleros 50, 05003, Ávila, Spain

[2]Department of Prehistory, Complutense University, Prof. Aranguren s/n, 28040, Madrid, Spain

[3]C. A. I. Archaeometry and Archaeological Analysis, Complutense University, Professor Aranguren 2/n, 28040, Madrid, Spain

[4]Department of Prehistory and Archaeology, UNED University. Humanities Faculty, C/ Senda del Rey, 7, 28040, Madrid, Spain

[5]Institut Català de Paleoecologia Humana I Evolució Social (IPHES), Zona Educacional 4, Campus Sescelades URV (Edifici W3), 43700, Tarragona, Spain

[6]Universitat Rovira i Virgili (URV), Department d'Historia I Hostiora de l'Art, Avignuda de Catalunya 35, 43002, Tarragona, Spain

[7]Unit Associated to CSIC, Departamento de Paleobiologia, Museo de Ciencias Naturales, calle José Gutiérrez Abascal, s/n, 28006 Madrid, Spain

[8]Department of Biology, Zoology Unit, Autónoma University of Madrid, C/ Darwin 2, Campus Universitario de Cantoblanco, 28049, Madrid, Spain

[9]Center of Investigation in Biodiversity and Global Change (CIBC-UAM), Universidad Autónoma de Madrid, Madrid, Spain.

[10]Department of Topographic and Cartography Engineering, Higher Technical School of Engineers in Topography, Geodesy and Cartography, Technical University of Madrid, Mercator 2, 28031, Madrid, Spain

[11]Gran Duque de Alba Institution, Dibutación Provincial de Ávila, Paseo Dos de Mayo, 8, 05001, Ávila, Spain

[12]Department of Sciences of Communication and Sociology, Faculty of Communication Sciences, University Rey Juan Carlos, Camino del Molino, s/n, 28943, Fuenlabrada, Madrid, Spain

[*]Corresponding author. Email: ladc1995@gmail.com

August 6, 2021

Contents

Supplementary File S2 **2**

Supplementary File S3 **5**

Supplementary File S4 **6**

Supplementary File S5 **7**

Supplementary File S6 **9**

References **10**

Supplementary File S2

Appendix A presents a brief experiment performed to test the resolution of information that can be extracted from carnivore tooth scores using a 3D approach, as opposed to the 2D cross sections derived from 3D models. Here we experimented with similar approaches as described by Courtenay et al. [1], using 5 fixed landmarks marking maximum length (LM1 & LM2), width (LM3 & LM4) and depth (LM5), followed by different sized semi landmark patches. This was then compared using Geometric Morphometrics with the original 2D 7-landmark model by Yravedra et al. [2] (See Main Text).

Similar to Courtenay et al. [1], we used a published dataset of dog and wolf tooth scores which are clearly differentiable through using a 2D 7-Landmark model [3]. For Geometric Morphometric comparisons, we tested the amount of information contained by the Principal Components Analysis (PCA), followed by calculations of Procrustes Distances obtained after Generalized Procrustes Analysis (GPA). Considering how the original 2D landmark model was able to clearly differentiate between wolf and dog tooth marks, we used a Multivariate Analysis of Variance to calculate the degree of statistical differences. Finally a rudimentary Linear Discriminant Analysis (LDA) was performed to calculate this separation accuracy. Results are described in Table S2.1.

Supplementary Table S2. 1: *Comparison of Landmark models used to process 2D cross sections and entire 3D tooth score morphologies via semi-landmark based computational models. LM = Landmarks; PC = Principal Components; Proc. = Procrustes; D = Distance; p = p-value; MANOVA = Multivariate Analysis of Variance p-Values; LDA = Linear Discriminant Analysis accuracy. Columns PC1-2 and PC1-10 describe the amount of morphological variance captured across the corresponding PC scores.* $p(H_0)$ *are the calibrated p-values to Probability of Null Hypothesis percentages.*

	N°LM	PC1-2	PC1-10	Proc. D(p)	Proc. $p(H_0)$	MANOVA	$p(H_0)$	LDA
2D	7	80.6%	100.0%	0.13(0.0007)	1.4%	4.7e-05	0.1%	87%
3D	30	79.7%	96.7%	0.04(0.5)	51.5%	0.6	54.6%	40%
3D	41	79.8%	96.5%	0.04(0.5)	51.5%	0.7	59.6%	43%
3D	54	81.7%	97.0%	0.04(0.4)	50.1%	0.5	38.5%	53%

As can be seen, the 2D 7-landmark models originally described by Yravedra et al. [2] present the highest degree of resolution for the study of tooth scores. 3D models on the other hand create noisy datasets without clear separation between carnivore taxa, while the accuracy of standard LDA models falls by 34 to 47%.

To provide a brief discussion on the reasons behind this, the physics behind the creation of a tooth score are very different than those produced in other types of carnivore bone surface modifications. Tooth scores are produced by the dragging of tooth cusps across the surface of bone. Most descriptions of tooth score morphologies thus describe these marks as;

- "A scarring of the surface that is commonly linear... being deeper on parts of short curvature and shallower on surfaces with long curvature...". **Binford, 1981; Pg. 46-48** [4]
- "As teeth scrape compact bone, concentric layers of tissue (bone lamellae) are broken through, and the groove produced is seldom flat-walled and smooth... many carnivore tooth marks are made by the teeth of adult animals, which have relatively blunt or large cusps. These marks are characterized by uneven edges and incomplete slicing through bone lamellae". **Haynes, 1983; Pg. 165-166** [5]
- "U-shaped cross-sections... that commonly show crushing that is conspicuous under the hand lens". **Blumenschine, 1994; Pg. 29** [6]
- "Linear marks with U shaped cross-section... the marks left by teeth are often more abrasive, for their cutting edges are less sharp than those of stone tools". **Fernández-Jalvo & Andrews, 2016; Pg. 31** [7]

From these classic and detailed descriptions, alongside our own in-depth observations of tooth score morphologies both in experimental and archaeological situations, it can be seen that a common attribute of tooth scores are their irregular morphologies. From this perspective, the base and walls of marks are seldom smooth, product of the tooth tearing up cortical lamellae, combined with the added implication of any friction produced in the process. From this perspective, tooth scores are likely to present wide ranges of different morphologies, a property which requires very large sample

sizes in order to truly capture their nature, which will otherwise prove difficult to model. Considering how cross-sections capture information from very precise points, they are less likely to be distorted by these variations. Likewise, the smaller number of landmarks (7) decrease the likelihood of capturing too much information, providing a means of describing their general attributes, such as width and depth.

Supplementary File S3

Supplementary File S3 provides the three primary equations used to calculate robust deviations as opposed to more traditional calculations of a distributions standard deviation. Biweight Midvariance (BWMV) values are typically reported as their square root.

$$BWMV = \frac{n \sum_{i=1}^{n} a_i \left(x_i - \tilde{x}\right)^2 \left(1 - U_i^2\right)^4}{\left(\sum_{i=1}^{2} a_i \left(1 - U_i^2\right)\left(1 - 5U_i^2\right)\right)^2} \tag{S3.1}$$

$$a_i = \begin{cases} 1, & if\ |U_i| < 1 \\ 0, & if\ |U_i| \geq 1 \end{cases} \tag{S3.2}$$

$$U = \frac{x_i - \tilde{x}}{9\left(\tilde{x}\left(|x_i - \tilde{x}_x|\right)\right)} \tag{S3.3}$$

Supplementary File S4

Supplementary File S4 provides the three equations and parameters used for any Bayesian modelling described in the text. Figure S4.1 additionally summarizes equations S4.1 through to S4.4 visually in the form of a Kruschke diagram.

$$\mu \sim U\left(l = 0,\ h = 2\right) \tag{S4.1}$$

$$\sigma \sim \left|\mathcal{N}\left(0,\ \sigma_{\sigma} = 10\right)\right| \tag{S4.2}$$

$$\nu \sim Exp\left(\lambda = 1/20\right) \tag{S4.3}$$

$$y \sim \mathcal{T}\left(\mu,\ \sigma,\ \nu\right) \tag{S4.4}$$

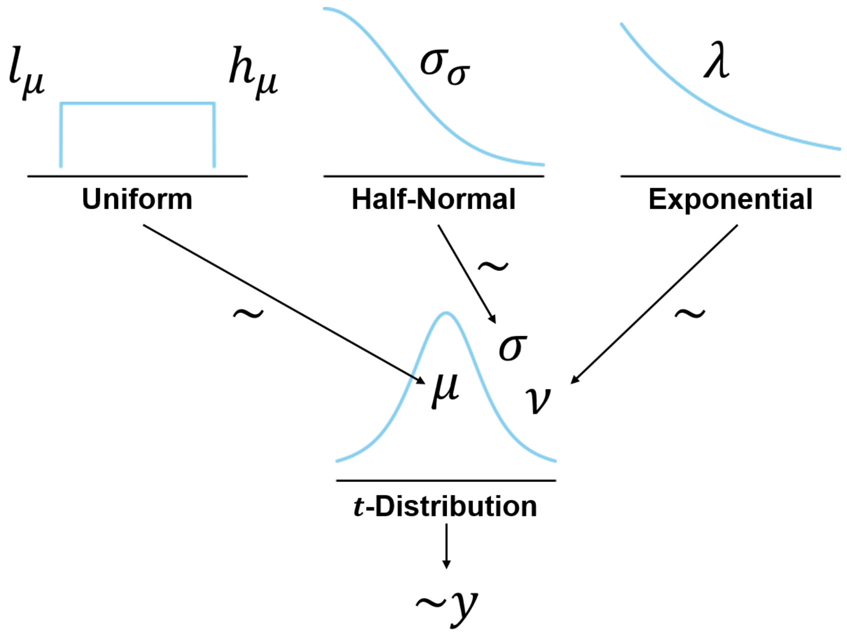

Supplementary Figure S4. 1: *Kruschke diagrams visually representing equations S4.1 to S4.4 for Bayesian inference calculations. Templates for Kruschke diagrams were provided by Rasmus Baath (`http://www.sumsar.net/blog/2013/10/diy-kruschke-style-diagrams/`)*

Supplementary File S5

Supplementary File S5 provides the equations for p-value callibrations as described by Benjamin and Berger [8] and Colquhoun [9], with slight adaptations in Equations S5.4 & S5.5 for the purpose of the present study. Tables S5.1 to S5.4 as well as Figure S5.1 additionally provide calibrations for a wide array of different p-values.

$$BF \leq BFB \equiv \frac{1}{-e\ p\ \log(p)} = L_{10} \tag{S5.1}$$

$$P^U(H_a|p) = \frac{BFB(p)}{1 + BFB(p)} \tag{S5.2}$$

$$FPR = \frac{1}{1 + L_{10}\frac{P(H_a)}{1-P(H_a)}} \tag{S5.3}$$

$$IFPR = \frac{1}{1 + L_{10}\left(1 - \left(\frac{P(H_a)}{1-P(H_a)}\right)\right)} \tag{S5.4}$$

$$P(H_0) = \begin{cases} FPR(p), & if\ p \leq 0.3681 \\ 1 - IFPR(p), & if\ p > 0.3681 \end{cases} \tag{S5.5}$$

Supplementary Table S5. 1: *Bayes Factor Bounds (BFB, eq. S5.1), Posterior Probability of H_a values ($P^U(H_a|p)$, eq. S5.2), Posterior Odds of H_a to H_0 values ($H_a : H_0$), and False Positive Risk (FPR, eq. S5.3) values, for a number of their corresponding p-values. Prior odds for $H_a : H_0$ and FPR calculations for the present table are set at 5:10 (0.5)*

p	0.368	0.200	0.100	0.050	0.010	0.005	0.003	0.001	0.0001	0.00001
BFB	1.000	1.140	1.598	2.456	7.988	13.89	21.11	53.26	399.4	3195
$P^U(H_a\|p)$	0.500	0.533	0.615	0.710	0.889	0.933	0.955	0.982	0.998	1.000
$H_a : H_0$	0.500	0.571	0.799	1.220	3.994	3.943	10.55	26.63	199.7	1598
FPR	0.500	0.467	0.385	0.289	0.111	0.067	0.045	0.018	0.002	0.0003

Supplementary Table S5. 2: *Bayes Factor Bounds (BFB, eq. S5.1), Posterior Probability of H_a values ($P^U(H_a|p)$, eq. S5.2), Posterior Odds of H_a to H_0 values ($H_a : H_0$), and False Positive Risk (FPR, eq. S5.3) values, for a number of their corresponding p-values. Prior odds for $H_a : H_0$ and FPR calculations for the present table are set at 7:10 (0.7)*

p	0.368	0.200	0.100	0.050	0.010	0.005	0.003	0.001	0.0001	0.00001
BFB	1.000	1.140	1.598	2.456	7.988	13.89	21.11	53.26	399.4	3195
$P^U(H_a\|p)$	0.300	0.329	0.406	0.513	0.774	0.856	0.900	0.958	0.994	0.999
$H_a : H_0$	0.700	0.800	1.118	1.719	5.592	9.721	14.77	37.28	279.6	2237
FPR	0.300	0.272	0.212	0.149	0.051	0.030	0.020	0.008	0.001	0.0001

Supplementary Table S5. 3: *Bayes Factor Bounds (BFB, eq. S5.1), Posterior Probability of H_a values ($P^U(H_a|p)$, eq. S5.2), Posterior Odds of H_a to H_0 values ($H_a : H_0$), and False Positive Risk (FPR, eq. S5.3) values, for a number of their corresponding p-values. Prior odds for $H_a : H_0$ and FPR calculations for the present table are set at 3:10 (0.3)*

p	0.368	0.200	0.100	0.050	0.010	0.005	0.003	0.001	0.0001	0.00001
BFB	1.000	1.140	1.598	2.456	7.988	13.89	21.11	53.26	399.4	3195
$P^U(H_a\|p)$	0.700	0.727	0.788	0.851	0.949	0.970	0.980	0.992	0.999	1.000
$H_a : H_0$	0.300	0.343	0.479	0.737	2.397	4.166	6.333	15.98	119.8	958.6
FPR	0.700	0.671	0.593	0.487	0.226	0.144	0.100	0.042	0.006	0.0007

Supplementary Table S5. 4: *$p(H_0)$ values (eq. S5.5) for a number of their corresponding p-values using different priors. Values are visually represented in Fig. D1*

p	0.990	0.800	0.500	0.368	0.100	0.050	0.010	0.005	0.003	0.001
0.3 Prior	0.989	0.828	0.712	0.700	0.594	0.487	0.226	0.144	0.100	0.042
0.5 Prior	0.974	0.673	0.515	0.500	0.385	0.289	0.111	0.067	0.045	0.018
0.7 Prior	0.941	0.469	0.313	0.300	0.212	0.149	0.051	0.030	0.020	0.008

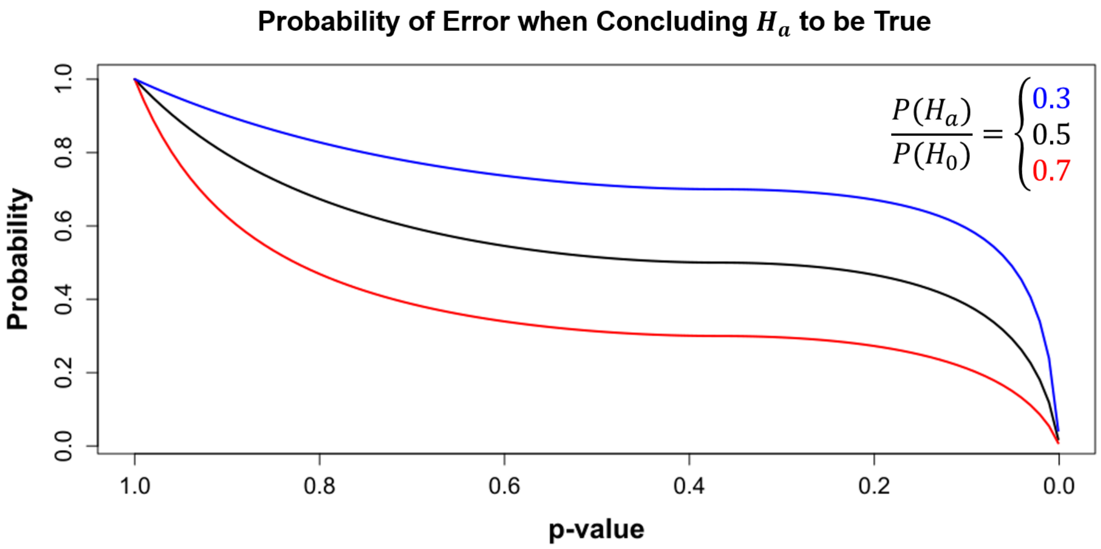

Supplementary Figure S5. 1: *Visualization of calibration curves for p-values and the probability of reporting an error when concluding the alternative hypothesis to be true ($p(H_0)$).*

Supplementary File S6

Supplementary Table S6. 1: *Frequency of taphonomic long bone modifications observed in each of the samples. Preliminary data has been derived from more developed research pending publication [10].*

	Sample Size	Bones with Tooth Marks (%)	Bones presenting salivary alterations (%)	Epiphysis Survival (%)	Average Number of Tooth Marks per Specimen
Villardeciervos*	99	90.4	41.7	89.8	19.5
Flechas*	46	100.0	67.4	85.9	27.7
Cabárceno	23	100.0	85.4	45.0	81.0
Hosquillo	420	91.0	67.8	39.0	16.5

* Wild animal samples

Supplementary Table S6. 2: *Description of bones presenting less than 50%, more than 50%, and the entirety of their total circumference. Preliminary data has been derived from more developed research pending publication [10].*

	< 50%	> 50%	100%
Villardeciervos*	5 (5.1%)	2 (2.0%)	91 (92.9%)
Flechas*	0 (0.0%)	0 (0.0%)	46 (100.0%)
Cabárceno	0 (0.0%)	1 (4.4%)	22 (71.8%)
Hosquillo	284 (67.8%)	60 (14.3%)	75 (0.95%)

* Wild animal samples

Supplementary Table S6. 3: *Description of bones presenting less than 25%, between 20 to 50%, more than 50%, and the entirety of their total length. Preliminary data has been derived from more developed research pending publication [10].*

	< 50%	25-50%	> 50%	100%
Villardeciervos*	15 (15.2%)	3 (3.0%)	42 (42.4%)	39 (39.4%)
Flechas*	1 (2.2%)	0 (0.0%)	12 (26.1%)	33 (71.7%)
Cabárceno	0 (0.0%)	0 (0.0%)	0 (0.0%)	8 (100.0%)
Hosquillo	143 (34.1%)	209 (49.8%)	64 (15.2%)	4 (1.0%)

* Wild animal samples

References

[1] L. A. Courtenay, D. Herranz-Rodrigo, R. Huguet, M. Á. Maté-González, D. González-Aguilera, and J. Yravedra, "Obtaining new resolutions in carnivore tooth pit morphological analyses: A methodological update for digital taphonomy," *PLoS ONE*, vol. 15(10), e0240328, 2020. DOI: 10.1371/journal.pone.0240328.

[2] J. Yravedra, E. G. Vargas, M. Á. Maté-González, J. Aramendi, J. Palomeque-González, J. Vallés-Iriso, J. Matesanz-Vicente, D. González-Aguilera, and M. Domínguez-Rodrigo, "The use of micro-photogrammetry and geometric morphometrics for identifying carnivore agency in bone assemblage," *Journal of Archaeological Science: Reports*, vol. 14, pp. 106–115, 2017. DOI: 10.1016/j.jasrep.2017.05.043.

[3] J. Yravedra, M. Á. Maté-González, L. A. Courtenay, D. González-Aguilera, and M. Fernández-Fernández, "The use of canid tooth marks on bone for the identification of livestock predation," *Scientific Reports*, vol. 9, p. 16 301, 2019. DOI: 10.1038/s41598-019-52807-0.

[4] L. R. Binford, *Bones: Ancient Men and Modern Myths*. New York: Academic Press Inc, 1981.

[5] G. Haynes, "A guide for differentiating mammalian carnivore taxa responsible for gnaw damage to herbivore limb bones," *Paleobiology*, vol. 9(2), pp. 164–172, 1983.

[6] R. J. Blumenschine, "Percussion marks, tooth marks and the experimental determinations of the timing of hominid and carnivore access to long bones at FLK Zinjanthropus, Olduvai Gorge, Tanzania," *Journal of Human Evolution*, vol. 29, pp. 21–51, 1995.

[7] Y. Fernández-Jalvo and P. Andrews, *Atlas of Taphonomic Identifications*. The Netherlands: Springer, 2016.

[8] D. J. Benjamin and J. O. Berger, "Three recommendations for improving the use of p-values," *The American Statistician*, vol. 73(Sup1), pp. 186–191, 2019. DOI: 10.1080/0031305.2018.1543135.

[9] D. Colquhoun, "The False Positive Risk: A proposal concerning what to do about p-values," *The American Statistician*, vol. 73(Sup1), pp. 192–201, 2019. DOI: 10.1080/00031305.2018.1529622.

[10] J. M. Vázquez-Rodríguez, *Estudio actualista del lobo como agente tafonómico: implicaciones de su comportamiento para la interpretación de yacimientos arqueológicos*. Madrid: UNED, Unpublished.
